# Supplementary material for: Three-dimensional view of ultrafast dynamics in photoexcited bacteriorhodopsin
Source: Nat Commun. 2019 Jul 18;10:3177. doi: 10.1038/s41467-019-10758-0 (PMC6639342; doi:10.1038/s41467-019-10758-0)
Supplement: Supplementary file 1 — Supplementary Information [file 41467_2019_10758_MOESM1_ESM.pdf]

Supplementary Information

Three-dimensional view of ultrafast dynamics in photoexcited bacteriorhodopsin

G. Nass Kovacs et al.

SUPPLEMENTARY MATERIAL

- **SUPPLEMENTARY METHODS**
- **SUPPLEMENTARY NOTES (1, 2)**
- **SUPPLEMENTARY FIGURES (1-23)**
- **SUPPLEMENTARY TABLES (1-10)**

SUPPLEMENTARY METHODS

Calculation of electron density maps

Time delays

Pump-probe data were collected at nominal time delays of 0.5, 1, 3 and 10 ps, as well as at a time delay of 33 ms. During collection of the 0.5 and 1 ps time delay data, a timing tool[1] was used to determine the actual delay between the arrival of the pump laser and FEL pulses. Data collected at time delays of 1 ps and longer were used as-is, all 1 ps time delay data was binned into a single data set. However, the 0.5 ps time delay data contained frames collected with time delays between 100 and 800 fs, because of the jitter in the difference between the FEL- and pump laser arrival times of ~300 fs.
Therefore, all ~43,000 images in the nominal 0.5 ps dataset were sorted by actual time delay as determined by the timing tool. Then, the data were binned into 12 datasets of 10,000 images each, with average time delays of 0.24, 0.33, 0.39, 0.43, 0.46, 0.49, 0.53, 0.56, 0.59, 0.63, 0.68 and 0.74 ps. There is considerable overlap between the datasets; the standard deviation, as well as the minimum and maximum time delays in each dataset are shown in Supplementary Figure 18. We cannot exclude that the larger time spread in the later sub-ps time-delay bins causes damping of the observed oscillations in distances and torsion angles.

Indexing ambiguity and twinning

The indexing ambiguity present in the data was resolved using the software module ambigator[2,3] and the results were checked by inspecting the self-rotation function of each data set as calculated by molrep[4] and checked for twinning using the detwin program[5]. Both methods check for the presence of rotational symmetry that should not be present given the actual space group symmetry, in this case 6/mmm point group symmetry despite the $P6_3$ space group. Whereas the self-rotation function indicated only negligible amounts of residual 6/mmm character, xtriage[6] indicated small twinning fractions of at most 10.1% (for the 0.63 ps time point) as judged by the maximum likelihood criterion. Possibly, this indicates a small amount of 'real' twinning in the crystals. However, accounting for twinning did not result in better R-factors in refinement nor in higher peaks in the pumped-unpumped difference maps, and the residual 6/mmm symmetry was therefore ignored.

Interestingly, calculating a self-rotation function for the Nango *et al.* dark-state data[7] shows strong peaks in the 180° slice consisted with 6/mmm symmetry (peak height 94% of the origin

peak), although detwin[8] and, as reported by the authors, xtriage[6], indicate no significant twinning for these data. This puzzling result suggests that the normal means of estimating twinning fractions may be compromised in the case of Monte-Carlo-integrated SFX data, possibly because of their intensity statistics.

Anisomorphism

During the experiment, it became apparent that at least two distinct crystal forms were present in different crystal batches, varying slightly in the length of their unit cell axes, and that some data sets were collected from the one crystal form and some from the other. This property of bR crystals has been suggested to give rise to spurious results and lead to misinterpretation of time-resolved data[9]. It was therefore necessary to collect reference (unpumped) data from both crystal forms and refine two reference structures independently, and to match the correct reference data and phases to each time point so as to avoid contaminating the maps with the effects of anisomorphism.

Difference density maps

q-weighted difference electron density maps were calculated according to[10,11]. Briefly, Monte-Carlo integrated intensities were converted to structure factor amplitudes using truncate[8] and scaled to the reference (preilluminated, unpumped) data using scaleit (including Wilson scaling)[8]. Weights for the q-weighting were calculated with custom-written python scripts. The amplitude differences were further weighted with the figure-of-merit (FOM) weight m from the refinement used to obtain the reference unpumped structure, to account for the uncertainty in the phase angles. Both q- and FOM-weighted as well as FOM-only weighted maps were inspected in coot[12,13]. However, the difference electron density maps were difficult to interpret in structural terms. We therefore calculated 'extrapolated' electron density maps, in which the occupancy of the pumped state is extrapolated to 100% (see below).

Extrapolated density maps (Method 1)

Extrapolated structure factors were calculated according to[14,15]:

$|F_{extrapolated}| = \frac{(|F_{pumped}|-|F_{unpumped}|)}{f} + |F_{unpumped}|$, where f is the expected occupancy of the pumped state. This method, called "scalar approximation of structure factor extrapolation", assumes that there is no phase difference between F_{pumped} and $F_{unpumped}$, and therefore only works reliably for small differences between the structures.

A recent light-triggered time-resolved crystallography study[16] reported the use of calculated structure factors of the unpumped state ($F_{calc,dark}$) for the extrapolation of structure factor amplitudes to full occupancy of the pumped state, rather than the observed amplitudes ($F_{obs,dark}$). However, any error in those calculated amplitudes, be they due to an error in the atomic positions, to an inadequate bulk solvent model or to something else, will thus be introduced into the extrapolated structure factors. We therefore used the observed amplitudes for structure factor extrapolation.

When calculating extrapolated structure factors, the choice of f has a large impact on the electron density maps; when the value is too low, the features of the pumped state are

exaggerated, whereas a too high value results drowns out the features. We therefore calculated maps for the 33 ms state, for which the expected features are well known, while varying f in steps of 0.05 starting from 0.05 and inspected the maps. The occupancy was then determined as the highest value of f where no signs of the unpumped structure were apparent. For the 33 ms time point, the electron density of Asp212, Arg96 and Trp182 were monitored. At values of f higher than 0.15, the density for the side chains of these residues showed clear contributions of the dark state. As there were no strong features to decide on the value of f for the sub-ps and 1 ps time delays, and since these were collected under similar circumstances, we chose 0.15 as the value for f for these time points as well. Maps calculated at $f = 0.20$ were very similar to those calculated at $f = 0.15$, albeit with smaller deviations from the dark-state structures. For the 3 ps data, the decision was made based on the density for water molecule Wat402. From the difference density maps, it is clear that this molecule moves away from the Lys216 NZ atom. In extrapolated maps using $f = 0.10$, a single position for this water molecule is seen which is indeed shifted away from its dark-state position, whereas at $f = 0.15$, the density for this water became elongated, now also encompassing the dark state position. A similar observation was made for the C14 carbon atom of the retinal. We therefore used $f = 0.10$ for the 3 ps time point. The difference electron density maps for the 10 ps time delay showed clear evidence for a tilting of the C20 methyl group of the retinal towards Tyr185. We therefore used this feature to decide on the value of f; at $f = 0.15$, the map started to show the untilted, dark-state position of this methyl group, and we therefore used $f = 0.10$ for the 10 ps time delay data.

We evaluated the use of q-weighted differences in structure factor extrapolation. We found that, in maps calculated using $F_{extrapolated}$ and phases/FOMs from the most isomorphous dark-state structure, this results in the same features that were observed without q-weighting, but at larger values of f. As the results were similar, we did not use q-weighting for the extrapolation of structure factor amplitudes.

Singular value decomposition of electron density maps

To assess the information content of the extrapolated electron density maps, and to obtain information on the time course of events, we performed singular value decomposition[16,17] on the extrapolated maps ($f = 0.15$) for the 12 sub-ps time points. To this end, each three-dimensional map array of n voxels was linearized, and the resulting vectors for each of the m time points were combined into an $n \times m$ array of elements that was factorized as follows:

$$\begin{pmatrix} \rho(\overrightarrow{r_0}, t_0) & \dots & \rho(\overrightarrow{r_0}, t_m) \\ \dots & & \dots \\ \dots & & \dots \\ \dots & & \dots \\ \dots & & \dots \\ \rho(\overrightarrow{r_n}, t_0) & \dots & \rho(\overrightarrow{r_n}, t_m) \end{pmatrix} = U \cdot S \cdot V^{*T}$$

where:

$$U = \begin{pmatrix} U_0(\overrightarrow{r_0}) & \dots & U_n(\overrightarrow{r_0}) \\ \dots & & \dots \\ \dots & & \dots \\ \dots & & \dots \\ \dots & & \dots \\ U_0(\overrightarrow{r_n}) & \dots & U_n(\overrightarrow{r_n}) \end{pmatrix}, \quad S = \begin{pmatrix} S_0 & & \\ & \dots & \\ & & S_m \end{pmatrix}, \quad V^{*T} = \begin{pmatrix} V_0(t_0) & \dots & V_0(t_m) \\ & & \\ V_m(t_0) & \dots & V_m(t_m) \end{pmatrix}$$

In this way, the maps are decomposed into orthogonal "maps" $\begin{pmatrix} U_a(\overrightarrow{r_0}) \\ \dots \\ \dots \\ \dots \\ \dots \\ U_a(\overrightarrow{r_n}) \end{pmatrix}$ that contribute to the actual maps with a weight S_a and a time evolution described by $(V_a(t_0) \quad \dots \quad V_a(t_m))$.

When applied to the extrapolated maps for the 12 sub-ps time points, the diagonal elements of S were as shown in Supplementary Figure 10a. The first and strongest contribution corresponds to the average of the 12 original maps. The other contributions, 2-12, are far smaller, with contributions 2-5 probably containing signal and number 6-12 probably mainly containing noise.

We investigated the three strongest contributions, number 1-3, whose time evolution $V_a(t)$ is shown in Supplementary Figure 10b. As expected, the time evolution of the first contribution, the average, is virtually constant with time. The time course of the second contribution looks like a Gaussian centered at ~0.5 ps, and that of the third is reminiscent of a step function, centred also at ~0.5 ps. These features were indifferent to the value of f used for the structure factor extrapolation (not shown).

Investigation of the left singular vectors U_a by themselves is not straightforward. We therefore amplified the effects of the contributions 2 and 3 by multiplying their respective weights S_a by a factor of three and setting the other elements of S to zero, apart from the weight of the first contribution. *I.e.*, to investigate the effects of contribution number 3, a new matrix S^{ampl} was constructed as follows:

$$S^{ampl} = \begin{pmatrix} 1.0 & & & & & & \\ & 0 & & & & & \\ & & 3.0 & & & & \\ & & & 0 & & & \\ & & & & 0 & & \\ & & & & & \dots & \\ & & & & & & \dots \end{pmatrix} \cdot S$$

Then, a matrix containing 'amplified component' maps were reconstructed using:

$$\begin{pmatrix} \rho^{ampl}(\overrightarrow{r_0}, t_0) & \dots & \rho^{ampl}(\overrightarrow{r_0}, t_m) \\ \dots & & \dots \\ \dots & & \dots \\ \dots & & \dots \\ \dots & & \dots \\ \rho^{ampl}(\overrightarrow{r_n}, t_0) & \dots & \rho^{ampl}(\overrightarrow{r_n}, t_m) \end{pmatrix} = U \cdot S^{ampl} \cdot V^{*T}$$

Inspection of the 'amplified component' maps (not shown) around the retinal revealed that in addition to changes all over the protein, contribution number 2 shows the distortion at the C13-C14 bond seen in the original maps, and that contribution number 3 displays the 'bending' of the retinal that is contained in the original maps.

While these contributions by themselves should on no account be interpreted as separate species, modes or events, these results do establish that a major change occurs in the structure at around 0.5 ps.

Refinement of unpumped-state structures

Both structures for both forms of the unpumped state were obtained by refinement using phenix.refine[6], using default settings. For a better comparison of the retinal torsion angles in the dark state and in the time-resolved structures, an additional dark structure, termed dark-unrestrained was refined using exactly the same protocol as the time-resolved structures against a dataset generated from 10,000 images. For the refinement, the retinal geometry file was modified as described below in "Retinal geometry". Data and refinement statistics are given in Supplementary Table 4.

Refinement of ps and sub-ps time delay structures

The refinement of structures of low-occupancy populations is extremely challenging. Various methods exist, such as ensemble refinement, as was used in *e.g*[7], and refinement against extrapolated structure factors, as was used in *e.g*[16]. We evaluated both methods with our data, but found that the resulting structures contained many spurious deviations from the extrapolated electron density maps, likely due to the comparatively large errors introduced by the extrapolation algorithm, in addition to the inherently large error of structure factor amplitudes determined by SFX.

Since electron density maps are relatively insensitive to errors in the structure factor amplitudes, we chose to perform real-space refinement against the extrapolated electron density maps for the sub-ps and ps time delay data, starting each time from the most isomorphous unpumped structure as judged by the cell parameters. This resulted in much more stable refinement, and final structures that excellently matched the electron density maps. Real-space refinement was performed using phenix.real_space_refine[6,18] against maps calculated using $F_{extrapolated}$ and phases/FOMs from the most isomorphous dark-state structure, with f as indicated in Supplementary Table 4, which also contains the data and refinement statistics.

Orthogonal approaches to the refinement of sub-ps time delay structures (Method 2)

We sought to verify whether the selection of software used to generate extrapolated structures factors affects the outcome of the refinement. Extrapolated structure factors were thus generated for various occupancies of the transient structure, ranging from 10 to 33% using: $|F_{extrapolated}| = \alpha * (F_{pumped} - F_{unpumped}) + F_{unpumped}$, where α is the inverse of the occupancy. Differences between Method 1 and Method 2 included the software used to scale datasets and the bulk solvent model (respectively, the 'fom' and 'scalenbulk' modules of CNS in Method 2, instead of CCP4 'scaleit' in Method 1), to generate Q-weights (a custom-modified CNS module vs. custom-written python script), to calculate Q-weighted difference maps (CNS 'fo-fo_map.inp' in Method 2 vs. CCP4 'fft' in Method 1) and to refine structures (secondary structure restraints derived from the dark model were used, in Method 2). Extrapolated electron density maps were then generated as described above for Method 1, *i.e.* based on extrapolated structure factors and on the phases and figures of merits (m) from the dark model (Fourier coefficients of the maps : $m^{dark} * |F^{ext}| * \exp[i * \varphi^{calc}]$). The dark structure was then fitted into these maps, using real space refinement (RSR) as implemented in *phenix.real_space_refine*. For all structures at all α values, the map-to-model correlation coefficient (CC_mask) was found to increase after RSR. Furthermore, *a posteriori* calculation of R-factors (after bulk-solvent modelling, but without refinement of the structure in the reciprocal space) reveals that these diminish after RSR, again for all pump-probe delays and at all α values. Increasing the value of α nonetheless results in a degradation of extrapolated maps which, in turn, yields a lower CC_mask and a correspondingly higher R-factor – as expected since α not only multiplies the signal, but also the noise present in the data. The oscillations seen in the structures refined by Method 1 were reproduced in the structures refined by Method 2, regardless of the α values, highlighting the robustness of these features in the raw data (Supplementary Figure 19).

Refinement of the 33 ms time delay structure

For the 33 ms time delay data, the extrapolated electron density maps had large gaps in the loops on the surface of the protein, likely because the large differences between the 33 ms and unpumped structure cause the assumptions at the basis of the structure factor extrapolation algorithm to break down. Thus, real-space refinement was not feasible for this time delay and ensemble refinement was performed instead, using phenix.refine[6].

To this end, in the most isomorphous unpumped-state structure, the retinal was split into an A and a B residue, with occupancies of 0.85 and 0.15, respectively. The same was done for residues 81-92, 175-185, and 208-221 surrounding the retinal, for which clear differences with respect to the unpumped state could be seen in the q-weighted $|F_{pumped}| - |F_{unpumped}|$ electron density maps. Then, the structure was first refined against the observed structure factor amplitudes, allowing only the B residues to move, after which a final round of refinement was performed in which all atoms were allowed to move. Data and refinement statistics are given in Supplementary Table 4.

Retinal geometry

To accommodate the observed retinal distortions in the sub-ps and 1 and 3 ps time delay structures, the RET.cif file defining the geometry of retinal in the phenix library was modified as follows: the planarity restraints were adapted in such a way as to allow atoms that are far away from each other to have more freedom to move out of their common plane, whereas for atoms that are closer together the planarity restraint is stronger. For the 10 ps time delay structure, geometric parameters, including torsion angles, where obtained from QM structure optimized with the B3LYP/cc-pvdz-D3 method.

Torsion angles and atom-atom distances

Values for torsion angles and atom-atom distances for the 0.24-0.74 ps data sets were determined using a python script. This was performed for structures refined against maps calculated using $F_{extrapolated}$ and phases/FOMs from the most isomorphous dark-state structure using values of f ranging from 0.10 to 0.25. The shape of the curves (period, position of maxima and minima) obtained in this way was indifferent to the choice of f, but their vertical position and scale depended on the value of f used. The period of the oscillatory motions were then determined from the highest peak in a Lomb-Scargle periodogram[19,20]. In addition, selected torsion angles and distances were fitted with a damped sinusoid[21] in the Grafit Data Analysis Software (Version 7), using the equation

$$A \sin(2\pi t\widetilde{\nu c} + \phi)\, e^{-at} + b$$

where A is the amplitude, t is time, $\tilde{\nu}$ is the wavenumber, c is the speed of light, ϕ is the phase, a is the damping constant and b is the offset. Although a damped sinusoid could be fitted, negligible damping is indicated (see main text for details on damping). Both methods gave same results within the error.

Error estimates

To estimate the uncertainties in the torsion angles and atom-atom distances, we used a jackknife-type method. Starting from the 10,000 images in the 0.43 ps dataset, we prepared six datasets with 9,000 randomly chosen images. Six structures were then refined against these datasets. The standard deviations observed for the torsion angles and atom-atom distances in these structures were then used as estimates for the errors in these values. For the uncertainty in the time delays we used the standard deviation of the time delays present in each dataset.

Retinal extraction and High Pressure Liquid Chromatography (HPLC) separation

Spectroscopic methods for the determination of all-*trans* and 13-*cis* content were not compatible with the injection procedure (no possibility to obtain a difference spectrum from an illuminated and not illuminated sample). Therefore, for the purpose of internally comparing samples in order to determine which pre-illumination protocol yields the highest all-*trans* retinal content and its approximate relative amounts, retinal extraction and HPLC separation was best suited as it is compatible with the injection protocol and is robust and fast. Retinal extraction from purple membranes was performed as described earlier[22,23], with the following modifications for crystalline samples. 8 µl of LCP+F-127 paste with bacteriorhodopsin microcrystals was mixed thoroughly with 165 µl ice-cold 75 % (*w/w)* ethanol by vigorous

pipetting. After 2 min of intermittent vortexing and incubation on ice, 125 µl ice-cold hexane was added and vortexed for 2 min. Phase separation of the emulsion was performed by centrifugation (1 min, 4 °C, 21130 x g, Eppendorf Centrifuge 5424 R) and the retinal isomers in the upper hexane layer were immediately separated by HPLC on a 125 x 4.6 mm ID Prontosil 120-3-Diol normal phase column (CS-Chromatographie Service GmbH) equilibrated in 9:1 hexane:ethyl acetate and running at 1.5 mL/min flowrate. The 13-*cis* isomer elutes in the first peak and the all-*trans* isomer in the second peak, as confirmed by comparing the retention times with an all-*trans* retinal standard (Sigma, R2500). The relative quantities of both isomers were calculated using integrated peak areas and extinction coefficients at 360 nm[23].

Calculation of the average path length through the crystal

Our plate-shaped hexagonal bR microcrystals have an average width *a*, length *b*, and thickness *c* of 25 µm, 25 µm and 2.5 µm, respectively. The crystals are embedded in an LCP stream which is injected into the vacuum chamber where it intersects with the XFEL beam/optical pump laser beam. The orientation of the crystals in the X-ray beam can be obtained from the orientation matrix derived from the indexed diffraction patterns: the highly preferred orientation is with the *c*-axis perpendicular to the jet axis (y-axis in the CrystFEL coordinate system). The *c*-axis is perpendicular to the plane of the crystalline plates (see Supplementary Figure 20a, b). Treating the crystals as disks with radius r and thickness d, and assuming that the beam intersects the crystal's vertical rotation axis, the path length through a crystal can be calculated as a function of the rotation of the crystal around the vertical axis (angle α) and a vertical offset from the centre of the crystal *o* (Supplementary Figure 20c). The offset *o* results in an effective "width" of the crystal 2r' that can be calculated as:

$$2r' = 2\sqrt{r^2 - o^2}$$

The dependence of the path length *p* on the angle α differs for two cases; for small α (Supplementary Figure 20d), the path length depends only on α and the thickness *d*:

$$p = \frac{d}{\cos\alpha}$$

At angles exceeding a critical angle α^{crit} (Supplementary Figure 20e, f) the effective width *r'* comes into play and the maximum path length becomes the diagonal through the crystal at offset *o*. This critical angle depends on both *d* and *r'* as

$$\alpha^{crit} = \frac{\pi}{2} - arctan\frac{d}{r'}$$

In this case the path length depends only on α and the effective width *2r'* (Supplementary Figure 20e, f):

$$p = \frac{2r'}{\cos(\frac{\pi}{2} - \alpha)}$$

Numerical integration over the angle α and the offset o for r=12.5 µm and d=2.5 µm result in an average path length <p> of 5.9 µm.

Estimation of transition from one- to two-photon regime in the IR power titration

Due to Lambert-Beer's law, the pulse energy density decays exponentially within the sample, complicating to estimate the number of photons/retinal. We describe two approaches, first the calculation of the ratio of the average number of photons and retinals within the excited volume and second the sample excitation fraction at the crystal surface.

I) Average number of photons per retinal

The bR concentration in PM samples was c = 10 mM. One bR molecule contains one retinal, thus the number of retinals N_{ret} in the excited volume V, defined by the path length p = 25 μm and the area of the pump laser with diameter d = 220 μm (1/e^2), is:

$$V = \pi(d/2)^2 \cdot p = \pi(0.11{\cdot}10^{-3}\mathrm{m})^2 \cdot 25 \cdot 10^{-6}\ \mathrm{m}\ = 9.50 \cdot 10^{-10}\ \mathrm{l}$$

$$N_{ret} = c\ VN_A = 0.010\ \frac{\mathrm{mol}}{\mathrm{l}} \cdot 9.50 \cdot 10^{-10}\mathrm{l} \cdot 6.022 \cdot 10^{23}\frac{1}{\mathrm{mol}} = 5.72 \cdot 10^{12}$$

Next, we calculate the average number of photons (N_p) per retinal in the entire excited volume V:

$$R_{av} = N_p/N_{ret} = \frac{E_p}{h\nu} \cdot \frac{1}{N_{ret}}$$

where h is Planck's constant and ν the frequency of the excitation laser. For a wavelength of λ=535 nm (corresponding to ν = 5.60· 10^{14} s^{-1}) R_{av} = 1 requires a pulse energy of

$$E_p = h\nu \cdot N_{ret} = \ 3.73\ \cdot\ 10^{-19}J\ \cdot 5.72\ \cdot 10^{12} = 2.13\ \mu J$$

For E_p = 0.7 μJ and pulse duration τ = 130 fs, the value corresponding to a peak power density of 35 GW/cm^2 in the fs-UV/VIS and 28 GW/cm^2 in the fs-mid-IR experiments, the lowest values used in the "power titration" experiments, the average number of photons per retinal is about 0.3. The calculation of the average N_p / N_{ret} in the TR-SFX experiment is given in Supplementary Table 7.

II) Number of photons per absorption cross section at front surface.

If F is the pump pulse fluence, the photon density at the front surface of the crystal is expressed by $n = F/h\nu$. Then, the sample excitation rate, the number of photons per absorption cross section, can be evaluated by $R_{surf} = \sigma F/h\nu$.

For a peak power density I and a pulse duration τ, $F = I\ \tau$, so that $R = \sigma I \tau/h\nu$

In the present condition I= 28 GW/cm^2, τ = 130 fs, one finds a fluence of F= 3.64 mJ/cm^2.

The relation between the absorption cross section σ and the extinction coefficient ε= 45 600 M^{-1} cm^{-1} at 532 nm, it is

$$\sigma = \varepsilon \cdot 1000\ \cdot \frac{\ln 10}{N_A} = 45\ 600\ \cdot 1000\ \cdot \frac{\ln 10}{6.022\ \cdot\ 10^{23}} = 1.74\ \cdot 10^{-16}\ cm^2$$

With $E_p = h\nu = 3.73\ 10^{-19}$ J, it follows

$$R = 1.7.$$

This number is consistent with the fact that pump-probe experiments in the linear response regime are usually carried out for $F \leq 0.4$ mJ/cm^2. Since R scales with the power density, $F = I\,\tau$, i.e. the TR- experiments have R values in the order of ~35.

SUPPLEMENTARY NOTE 1

Estimation of pump light scattered off the jet derived from calculations and measurements

The purple bR microcrystals are injected into the XFEL beam embedded in a round LCP stream. The incident pump laser light can be reflected, (de)focussed, absorbed, scattered and transmitted. In the following, these effects are delineated.

1. ***Ray tracing calculations to determine reflection from a cylindrical sample jet***

Using geometrical optics one can calculate the reflectance of an incoming light beam at the interface between the jet and its surrounding medium as well as the refraction at the interface and thus the trajectory of the light path through the medium. The incoming pump laser beam (532 nm, focused to 99 µm $1/e^2$ with a lens of 300 mm nominal focal length leading to a Rayleigh range of ~15 mm) was approximated to be collimated in a region of few hundred micrometres surrounding the jet. Two-dimensional ray tracing calculations of a fan of rays within the vacuum chamber (refractive index 1.0) onto the jet (refractive index 1.42, measured for LCP at 532 nm) were thus performed on a collimated light beam perpendicularly impinging onto a 100 µm diameter circular jet cross section as shown in Supplementary Figure 21. The angle of incidence of the various rays onto the jet was determined and the reflection for each was calculated using Fresnel equations. Tracing 5001 equally spaced rays inside a fan of a collimated light beam onto the jet gives a mean reflectance of 5.7 %. Since a fan of rays overestimates the contribution of rays hitting the jet edges compared to a bundle of rays with circular cross section, the presented calculation is an upper bound on the mean reflectance of the incoming laser beam with circular cross section. The lower bound is given by the most central ray hitting the jet perpendicularly, resulting in 3 % reflectance.

2. ***Estimation of pump light scattered by the sample medium***

The cylindrical jet acts as a cylindrical lens (for paraxial rays, the effective focal length (EFL) of a sphere with radius R and refractive index n becomes EFL = nR/(2(n-1)) ~85 µm[24]) that causes the direct beam exiting the jet to diverge strongly. The large divergence after passing through the jet precludes transmission measurements of the direct beam through the jet, unless one either has a very large detector or integrates over a large scattering angle. Therefore to determine the loss of light by the LCP with crystals, we measured the transmission of light through a rectangular cuvette with the flat surface facing the incoming light beam perpendicularly. The cuvette was filled either with a glycerol solution, with LCP (60:40 monoolein:water), with LCP and F-127 mixed 4+1 or with crystal-loaded LCP and F-127 mixed 4+1. To compensate for transmission losses due to reflectance at the cuvette interfaces, LCP and LCP mixed with F-127 were referenced to a 200 nm filtered glycerol solution (65 % w/w) which has the same refractive index, and crystal-loaded LCP mixed with F-127 was referenced to LCP mixed with F-127 such that the effect of crystals was apparent.

In a Jasco V-760 Spectrophotometer the transmission spectrum of the above mentioned samples was measured between 750 nm and 200 nm. The baseline of the spectrophotometer was recorded without any cuvette in place (i.e. through air) before each measurement series. A

cuvette with 100 µm path length (106-0.10-40, Hellma Analytics) was filled with 10-40 µl of sample and measured against air as a reference. The maximum acceptance angle of scattered light was ≤12°.

At 532 nm, the measured loss in transmission of LCP and LCP mixed with F-127 was ~1-2 %. For crystals embedded in LCP mixed with F-127 the measured loss in transmission resulting from the combined effect of absorption and scattering by the crystals (already corrected for scattering by LCP) was ~22 %.

3. *Estimation of pump light scattered off the crystal surface*

To disentangle the absorption and scattering components of the signal when it passes through a dispersion of bR crystals, the crystals were bleached by UV irradiation. A transmission spectrum of the crystal-loaded LCP mixed with F-127 was measured between 750 nm and 200 nm prior to bleaching and after 30 min, 60 min, 120 min, 180 min, 240 min and 300 min of UV irradiation (lamp VL-215.C, LTF Labortechnik; 254 nm, 2 x 15W). Using a light microscope, we verified that only the colour and not the shape of the crystals or transparency of LCP was altered by UV light (Supplementary Figure 22a, b). Correspondingly, the retinal absorption peak in the spectrum decreased over time and eventually smoothed out completely while the protein peak remained stable (Supplementary Figure 22c). To exclude contribution from potential UV-radiation induced changes of LCP absorption, control experiments were performed with LCP only (with and without F-127) showing no differences in the absorption at 532 nm after UV illumination for the same irradiation time (Supplementary Figure 22d). These results allow quantifying the absorption and scattering components of the spectrum. Before bleaching the absorbance is determined by scattering and absorption:

$$A(0\text{ min}) = A_{abs} + A_{scatt}$$

After bleaching, the measured absorbance is only determined by scattering:

$$A(120\text{ min}) = A_{scatt}$$

The bleaching process is complete after 120 min of irradiation without any signs of sample dehydration, which was however observed visually at the edges of the sample after 240 min and completely altered the sample absorption spectra after 300 min of bleaching. While the scattering of the crystals in principle depends on their absorption and changes in the crystals' OD might lead to different scattering behaviour[25], this effect is very small/negligible compared to other uncertainties.

For a pump wavelength of 532 nm we measured A(0min) = 0.107 and A(120min) = 0.073, indicating that ~15 % of incoming photons are scattered by the crystals while ~8 % are absorbed.

4. ***Photons fraction of pump intensity lost due to scattering and reflection at interfaces***

Sections 1-3 show that at 532 nm pump laser wavelength and a sample thickness of 100 μm, the following processes direct intensity away from the protein inside the crystals:

1. ~15 % of incoming photons are scattered by the crystals (see section 3)
2. ~1-2 % of photons are scattered by the LCP (see section 2)
3. ~3-6 % of photons are reflected at the surface of the LCP jet (see section 1)

We conclude that in total approximately 20 % of incoming photons are not seen by the protein inside the crystals due to scattering and reflection at the various interfaces.

SUPPLEMENTARY NOTE 2

1. ***Effect of the active space selection on the retinal and Trp86 energies***

The 8 electrons 5 molecular orbitals (MOs) active space (8,5) is shown in Supplementary Figure 8. This small-size active space consists of only the MOs describing static electronic correlation of the states of our interest. The CASSCF excited-state energies obtained with such an active space are large as compared to those determined from the experimental absorption-band maxima because the small active space essentially does not account for dynamical correlation. E.g., the CASSCF(8,5)-SA5 energy of the retinal S_0-S_1 transition (at S_0-min) is 3.16 eV whereas experimental maximum of bR at 570 nm corresponds to 2.18 eV. However performing the subsequent XMCQDPT2 calculations decreases the computed energy to 2.27 eV (546 nm), significantly improving agreement with the experimental spectrum. By including in the active space the occupied MOs of the electron donor residues coupled to the retinal chromophore, we obtained, in addition to the retinal S_1 energy, the energies of the intermolecular charge-transfer (CT) states corresponding to excitations from the occupied MOs of the donor to the unoccupied MOs of retinal. Similar to the excited states of the retinal, the CASSCF energies of the CT states computed with a small active space are rather large and significantly decrease after the dynamical correlation is accounted for by the XMCQDPT2 treatment. It is important to note that the XMCQDPT2 calculations decrease the energies of the locally excited states and CT states by a different amount (i.e., the so-called differential dynamical correlation) resulting in state reordering in the XMCQDPT2 spectrum as compared to the CASSCF spectrum. In contrast to the locally excited states of the retinal, the CT states show a small transition dipole moment with respect to the ground state, and therefore, the CT states do not show up as absorption bands in the experimental spectrum. For this reason, the computed CT energies cannot be compared to the experimental energies to gain confidence regarding the computational approach. On the other hand, the fact that the CT states are not observed in the excitation spectrum is probably the reason why these states have not been implicated in the excited state dynamics of bR although the role of electron-donor residues in tuning the bR absorption maximum has been recognized indicating a strong electronic coupling between the donors and retinal and hence, a possible photoinduced electron transfer process. Predicting the CT energies with a reasonable accuracy could provide a basis for further experimental characterization of these states using transient absorption experiments. The major

concern regarding our calculations is whether the predicted CT energies obtained using a small active space (8,5) are too low (which would result in an overestimation of their role in the bR excited-state dynamics). To address this concern, we present here computational results demonstrating the effect of the active-space selection on the CT energies.

The calculations with various active spaces were performed for a small cluster model comprised of the retinal protonated Schiff base (RPSB), Trp86 and W402 (Supplementary Figure 23a). The cluster was prepared by removing atoms from the optimized geometries of our large cluster model shown in Fig. 3 in the main text. At the boundaries of the small cluster, C-C bonds were replaces by C-H bonds (1.08 Å) without performing additional geometry optimization. The small models were prepared for the S_0-min and CT(W86)-min geometries of the large cluster. Note that the geometries of the large cluster were optimized with the (TD)-DFT method and thus, they do not depend on the active space selection. Also note that removing a large part of the model also excluded from consideration intermolecular interactions with residues surrounding the retinal and Trp86, in particular interactions with the counterions Asp85 and Asp212. In the absence of the counterions, the retinal energy decreases whereas the CT energy increases. The XMCQDPT2 and XMCQDPT2' excitation energies were computed with various active spaces and numbers of states included in the CASSCF energy averaging. The largest tested active space (12,12) consisted of 12 electrons and 12 MOs. While the (12,12) active space is quite large, it is computationally affordable. In addition, we performed the second-order approximate coupled-cluster model CC2 calculations[26] using the Turbomole 7.0.1 software[27]. All calculations employed the cc-pvdz basis set. Computed energies and properties are listed in Supplementary Tables 8 and 9.

The (12,12) active space comprised of the retinal MOs has been previously routinely used in calculations of various rhodopsins focusing on the retinal properties[28]. In order to explicitly account for the retinal-Trp86 charge-transfer interactions, we included MOs of both chromophores, retinal and Trp, in the active space, using the following partitioning (Ret8,8; Trp4,4), (Ret6,6; Trp6,6) and (Ret4,4; Trp8,8). The partitioning influences dynamical electronic correlation taken into account for each chromophore, especially at the CASSCF level of theory: the larger the active space, the lower the CASSCF excitation energy. The same holds true for the energies of the chromophore radicals that appear in the CT states. The dependence of the CASSCF energies on the active space selection eventually defines how many states should be included in the CASSCF calculations in order to compute the CT energy. For instance, for the three considered (12,12) active spaces the CT(W86) state was the fifth, fourth or third state in the CASSCF energy spectrum at the S_0-min geometry. On the other hand, the XMCQDPT2 energies depend on the active-space selection to a lesser extent than the CASSCF energies. In line with that, the CT(W86) state was the third state (following the retinal S_1) in the XMCQDPT2 energy spectra at the ground-state optimized S_0-min geometry (Supplementary Figure 23b). We note that the increase of the CT excitation energy by the active space variation is caused by the ground-state stabilization. All tested (12,12) active spaces yielded consistent results, predicting the CT(W86) state being by about 1 eV higher in energy than the S_1 retinal. The large active spaces were compared to the minimum-size active space (Ret2,2; Trp2,0) consisting of the retinal and Trp86 HOMOs and the retinal LUMO, which predicted the S_1-CT(W86) energy gap of 1.11 eV (XMCQDPT2) or 0.89 eV

(XMCQDPT2’). The CC2 method predicted even a somewhat smaller S_1-CT(W86) energy of 0.49 eV.

At the CT(W86)-min geometry, the tested large and small active-spaces also provided rather consistent results (Supplementary Figure 23c). With the exception of (Ret8,8; Trp4,4), all computed spectra showed the CT state being the first excited state. Substituting the active retinal MOs by the Trp MOs lowers the CT excitation energy. The CC2 results are rather close to the results obtained with the (Ret6,6; Trp6,6) active space. According to (Ret2,2; Trp2,0), the CT and S_1 state are very close in energy, thus the small active space rather underestimates stabilization of the CT state as compared to the larger “balanced” (i.e. equally divided for the donor and acceptor) active space. Overall, the computed small energy variations due to the method selection have no effect on the conclusion that the CT(W86) state may become the first excited state upon its structural relaxation. Population of the CT(W86) state by the retinal photoexcitation is described by the S_1/CT(W86) state crossing along the reaction coordinate connecting the S_0-min and CT(W86)-min geometries (Supplementary Figure 23d). Notably, the minimum active-space XMCQDPT2 calculations do not show this crossing to be more energetically favourable than the large active-space XMCQDPT2 calculation or CC2 calculations. Therefore, we are safe to conclude that our approach to estimating the CT energies using the minim-size active space accounting for static electron correlation is reliable.

2. ***Comparison of the XMCQDPT2 and XMCQDPT2’ results***.

We used a rather large cluster model for which a lack of size consistency of the quantum-chemical method may become a source of errors. The XMCQDPT2 method is not size consistent, therefore a different formulation termed XMCQDPT2’ was suggested in the original paper[29]. XMCQDPT2’ applies the MP2-like expression for the double-excitations from the double-occupied inactive MOs to the external MOs eliminating the dominant energy contribution violating exact core-separability. We compared the results of the two methods for retinal and our two cluster models in Supplementary Table 10. With the (Ret12,12) active space, the larger the model the larger the retinal S_1 energy computed with the XMCQDPT2 energies. For the large cluster model and the (12,12) active space, the XMCDQPT2 energy of the retinal S_1 state is significantly larger than the XMCDQPT2’ energy. The difference between the results of the two theories becomes smaller or even vanishes if a small active space is employed. The comparison of the retinal S_1 energies computed with the (12,12) active spaces to the experimental value indicates that the XMCQDPT2’ energies are more accurate for the large model. In line with that, we present the XMCQDPT2’-CASSCF(12,12)-SA20 energies in Fig. 4 and discuss them in the main text. In the Supplementary Tables 1,2, 8-10, energies obtained with both theories are presented. Note that with the small active space (8,5), the XMCQDPT2 and XMCQDPT2’ excitation energies are rather similar.

3. ***XMCQDPT2-CASSCF(12,12)-SA20 calculations.***

In order to estimate the excited state energies of Trp86 observed experimentally, we computed as many low-lying excited states of the Trp86 manifold as possible. To this end, we selected the (Ret4,4; Trp8,8) active space (Supplementary Table 2), including the complete double-bond subsystem of the indole moiety. Similar results were obtained with the (Ret6,6, Trp6,6)

active space (not shown). The computed spectrum consisted of the following excited states: excited states of retinal, designated (Ret)S_x, excited states of Trp, designated (W)S_x, delocalized stated combining the retinal and Trp excitations, designated (Ret)S_x(W)S_y, the Trp-retinal radical pair state CT(W86), excited-states of the Retinal and Trp radicals, designated (Ret)D_x and (W)D_x, respectively, and delocalized states combining the retinal radical and Trp radical excited states, designated (Ret)D_x(W)D_y. The assignment of the states was carried out by the analysis of the QDPT natural MOs and visual inspection of the differences between the state electron densities. Some delocalized combination states were difficult to assign to the particular states of the singlet of radical manifolds; these states are indicated as (Ret)S_x(W)S_y and (Ret)D_x(W)D_y in Supplementary Table 2. Both, XMCQDPT2 and XMCQDPT2' energies were analysed; in the main text we refer to the XMCQDPT2' energies.

Supplementary Figures

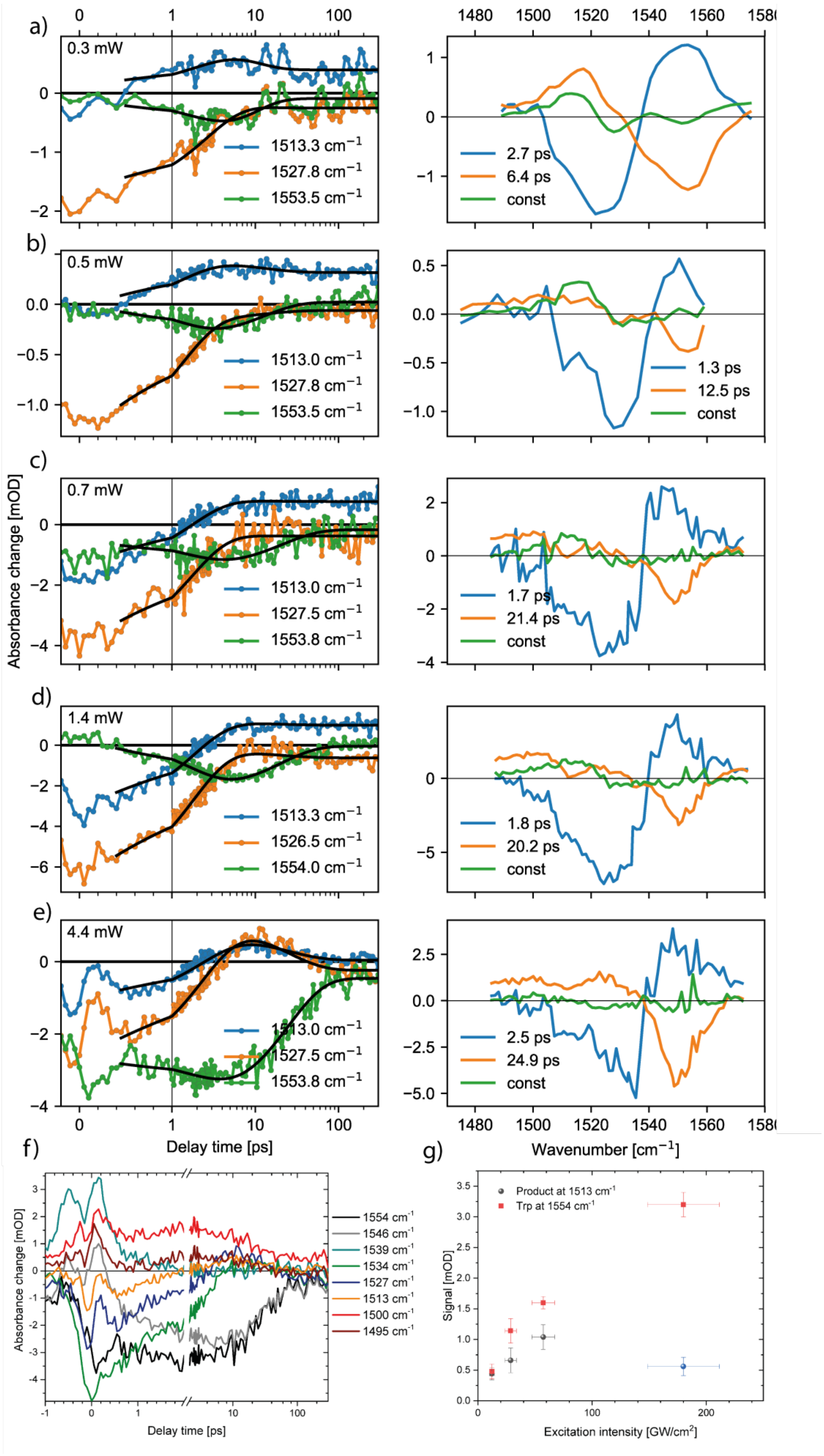

Supplementary Figure 1. a-e left panel: Transient dynamics and simulations (black lines) of bR in purple membranes excited at 535 nm with different excitation energies / intensities at frequencies of the product absorption at 1513 cm^{-1} (blue lines), the retinal C=C stretching bleaching signal at 1527 cm^{-1} (orange lines), and the Trp bleaching signal at 1554 cm^{-1} (green lines); **a-e right panel:** decay associated spectra of the dataset with a maximum of two decay time constants **a.** 0.3 µJ (12 GW/cm^2), **b,** 0.5 µJ (20 GW/cm^2), **c,** 0.7 µJ (28 GW/cm^2), **d,** 1.4 µJ (56 GW/cm^2), **e**, 4.4 µJ (180 GW/cm^2). Note, the experiments shown in **a** and **c-e** were performed under the same experimental conditions. The experiment in **b** at 0.5 µJ was performed after realigning the set-up, resulting in different absolute signal strengths. The main reason is the overlap of the pump and probe beams and the diameter of the beams. An additional sub-picosecond time constant was not used due to reduced signal-to-noise ratio at lowest excitation intensities. However, the sub-picosecond time constant is visible in the transients. **f,** transients for selected frequencies upon 180 GW/cm^2 excitation. **g,** dependence of the maximal product signal (circles) as a function of excitation intensity in the picosecond time range (10-20 ps) of the 1513 cm^{-1} transients; the transient at 180 GW/cm^2 is masked by emerging negative bands (compare transients in **f**) resulting in strongly reduced product signal (blue circle); dependence of the maximal Trp bleaching signal (red squares) as a function of excitation intensity in the picosecond time range (2-6 ps). The intensity dependence shows a sub-linear behaviour, with a linear range up to 30-60 GW/cm^2.

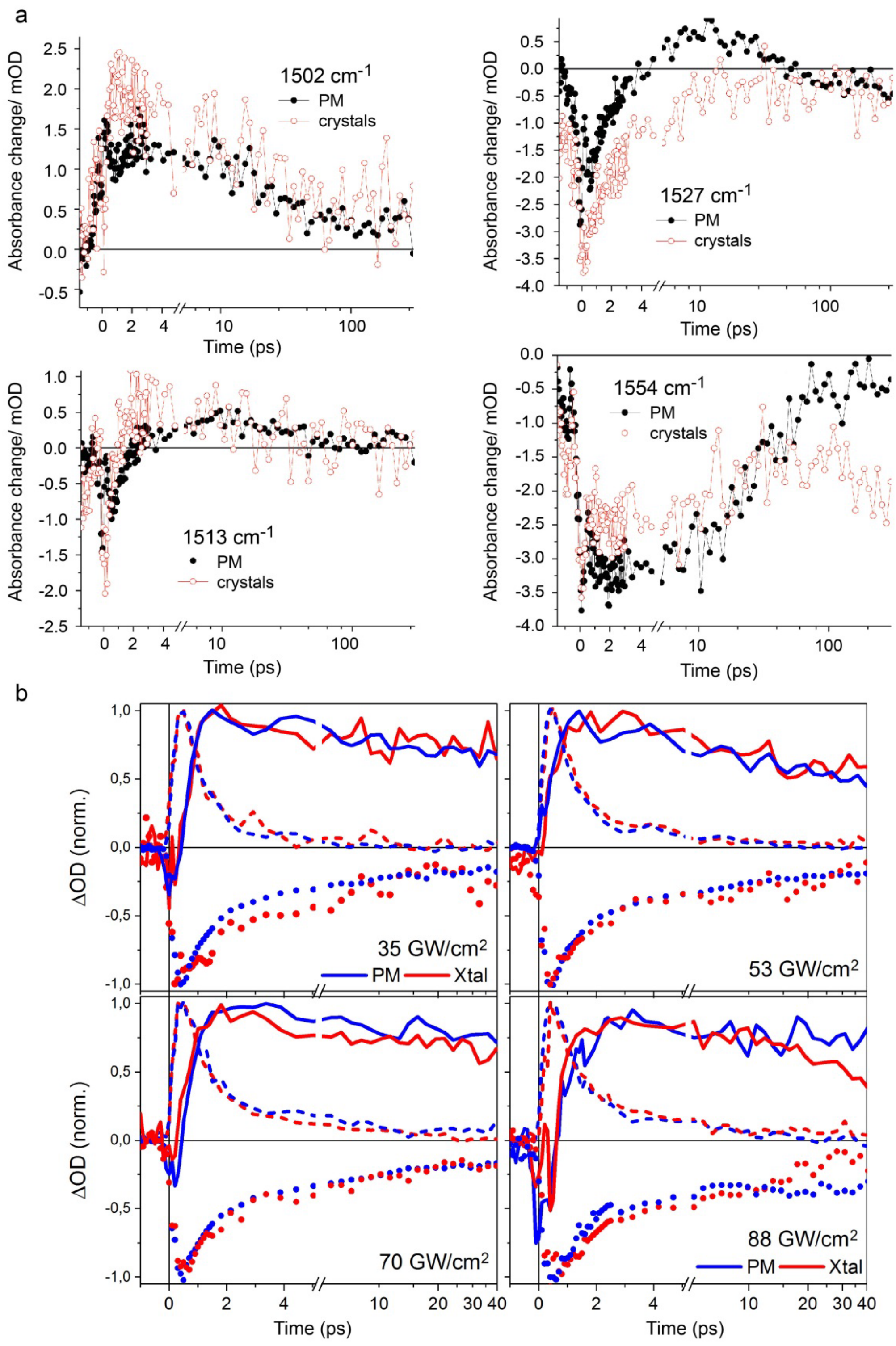

Supplementary Figure 2. a Direct comparison of bR transient dynamics in D_2O in purple membranes (PM) (black circles) and in microcrystals (red circles) at different wavenumbers at excitation energy of 4.4 µJ (180 GW/cm^2) upon 535 nm excitation. Transients at 1502 cm^{-1} (upper left) and 1513 cm^{-1} (lower left panel) show the same dynamics reflecting the same product formation in PM and microcrystals. No increase of product absorption is observed on the time-scale of tens of picoseconds, accompanied with the Trp decay presented in the lower right panel at 1554 cm^{-1}. Differences at 1527 cm^{-1} (upper right panel) between PM and microcrystals at about ten picoseconds are assigned to altered Trp relaxation pathways at high excitation energies. **b,** Direct comparison of kinetic traces in the UV/VIS region for

purple membrane (in H_2O, pH = 5.6) and the bR micro-crystals in LCP as a function of peak intensities (indicated in the panels). Within the present signal-to-noise ratio, the dynamics of 13-cis isomer formation (probe wavelength 670±5nm, solid lines), excited state decay (480±5 nm, dashed) and ground state recovery (570±5 nm, dots) are identical.

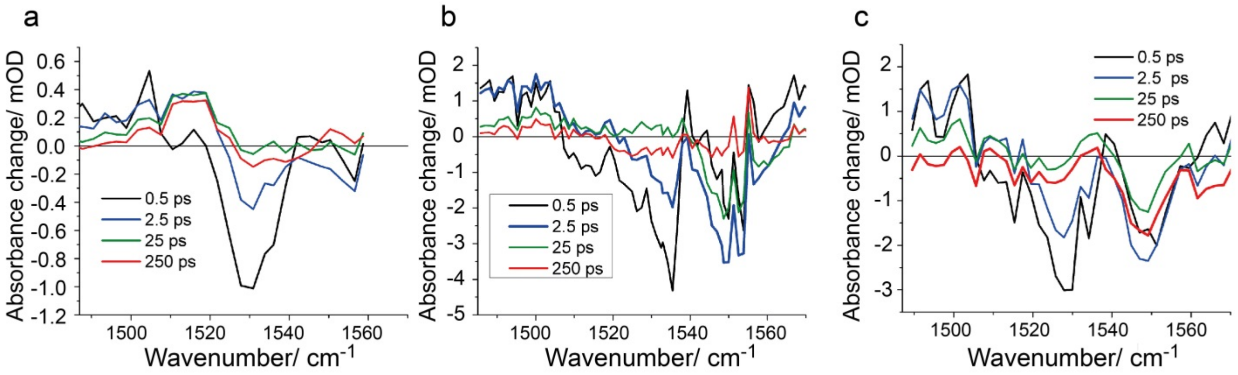

Supplementary Figure 3. Comparison of spectral dynamics upon excitation at 535 nm of bR in purple membranes (PM) at 0.5 µJ excitation energy (**a**), bR in PM at 4.4 µJ excitation energy (**b**), and bR microcrystals at 4.4 µJ excitation energy (**c**). At low energy the dynamics is dominated by the bleaching of the C=C stretching at 1527 cm^{-1} (see **a**). The bleaching signal at 1554 cm^{-1} of Trp is weak. With increasing energy the relative contribution of the Trp signal increases in PM (**b**) as well as in microcrystals (**c**). While the overall spectral shape changes with increasing energy, the transients of the retinal product formation at 1513 cm^{-1} and the retinal bleaching signal at 1527 cm^{-1} maintain nearly identical.

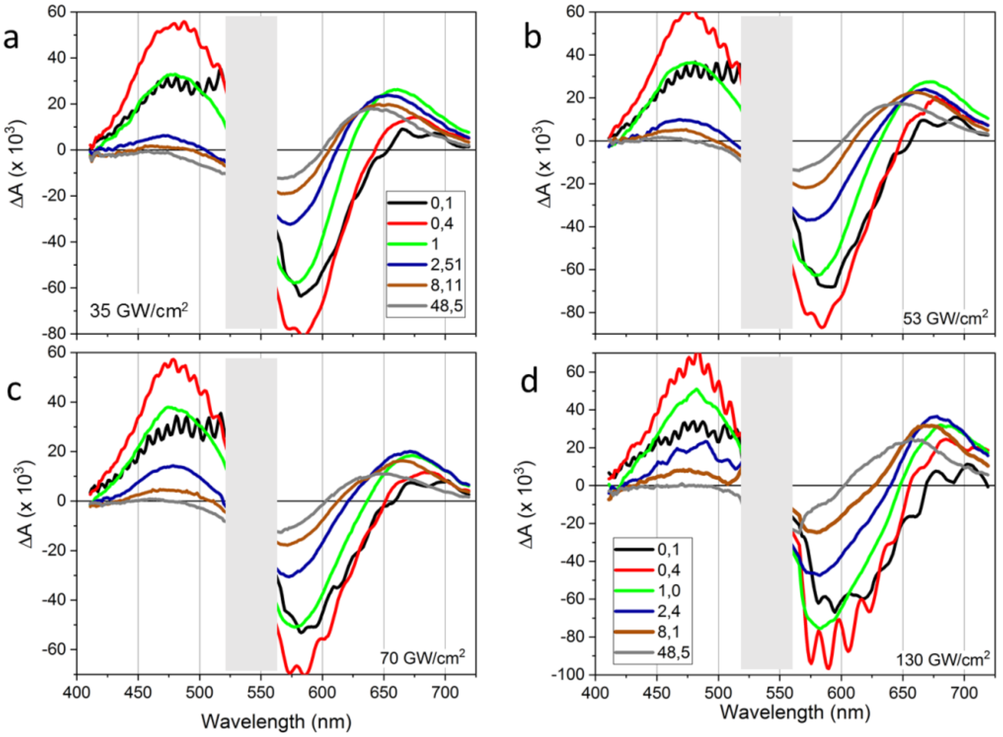

Supplementary Figure 4. Comparison of spectral dynamics in the VIS domain upon excitation at 535 nm of bR in purple membranes with a peak intensity of (**a**) 35, (**b**) 53 , (**c**) 70 and (**d**) 130 GW/cm^2. Delay times in ps are as indicated in the legend. Excited state absorption - ESA region: 420-520 nm. Ground state bleach - GSB: 550-620 nm, Photoproduct – PP: 600 – 720 nm. Data in the region 520-565 nm are affected by pump beam scattering. With increasing peak intensity, the PP band after 1.0 ps keeps its maximum in the 660-680 nm range, before relaxing to ca. 635 nm at 50 ps. This indicates that the usual J→K transition, occurring within 3-5 ps at low intensities (panel **a**) is overlaid by an additional absorption in the 660-680 nm range, the amplitude of which increases for higher intensities. We assign it to ESA of the S_1 state of Trp86. Another ESA, with similar temporal characteristics is observed as a weak signal centred at 470-480 nm for delay time ≥ 2 ps (panels **b-d**). Oscillatory features in the early delay time spectra are an artefact of the chirp correction procedure.

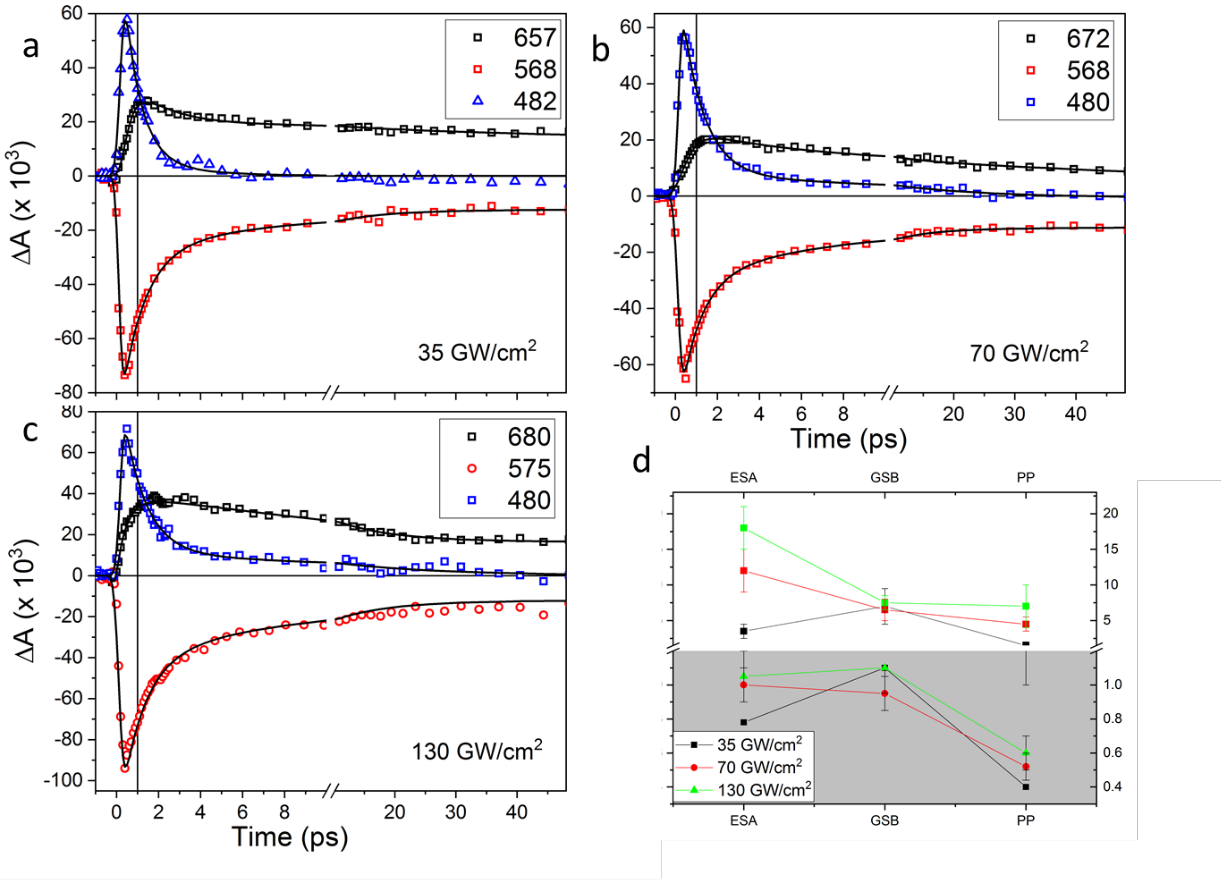

Supplementary Figure 5. Comparison of selected kinetic traces and their multi-exponential fits in the VIS domain upon excitation at 535 nm of bR in purple membranes with peak intensities of (**a**) 35, (**b**) 70 and (**c**) 130 GW/cm^2. Detection wavelength is as indicated in the legend, representative of ESA, GSB and PP (cf. Supplementary Figure 4). The appearance of the ESA of Trp86, due to a sequential two-photon absorption, is seen at 480 nm as a weak ~20 ps component in panels **b** and **c**. See also Fig. 2 in the main text. The PP kinetic traces are also significantly modified for higher pump intensities. While at 35 GW/cm^2 (panel **a**), a fast 1.5 ps decay component indicates the J→K transition, at 657 nm (the fitted time is wavelength-dependent), an additional rise of the signal and a slow ~10 ps decay, in particular for 130 GW/cm^2. **d** summarises the fitted lifetimes, probed in the three different spectral regions and their change with excitation intensity. The bottom part (grey) is representative of the all-*trans* → 13-*cis* isomerisation reaction. ESA, GSB and PP show an increase of the effective reaction time in agreement with previous publications[30,31]. The upper panel reveals the additional due to a sequential two-photon absorption-induced ~20 ps ESA lifetime, and a delayed spectral relaxation of the PP band (effective time scale 5-10 ps, for the wavelengths probed). The GSB times reflect the ground state recovery (formation of J state, ~1ps) and subsequent cooling (5 ps, for 568-575 nm) Note that, unlike for ESA and PP, the lifetimes in the GSB region are almost insensitive to the peak intensities, in line with the observations made from the mid-IR transient: Relaxation from of the Trp exc. state does not alter the all-*trans* → 13-*cis* isomerisation reaction kinetics.

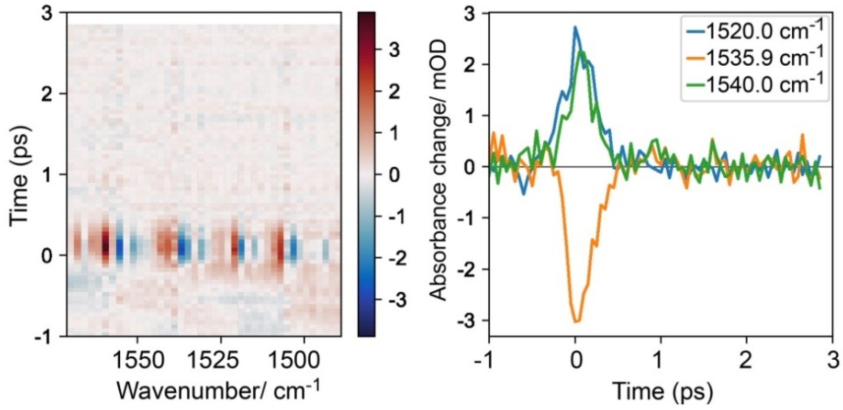

Supplementary Figure 6. Water dynamics upon excitation at 535 nm with 5.4 µJ excitation energy (220 GW/cm^2). In the complete spectral range from 1490 cm^{-1} to 1570 cm^{-1} signals are only visible during the pump-probe pulse overlap. These non-linear signals (e.g. cross-phase modulation signals) reflect the system-response of the experiment. No dynamics due to ionized water is observed in this spectral range.

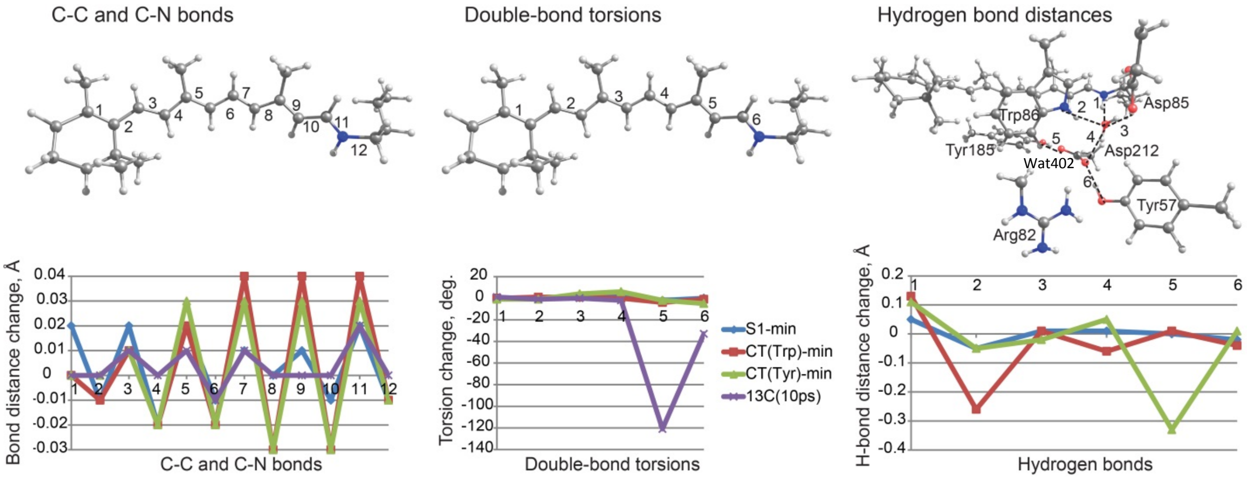

Supplementary Figure 7. Changes of the excited-state all-*trans* geometries S_1-min, CT(W86)-min and CT(Y185)-min and the ground-state 13-*cis* geometry S_0(10ps)-min with respect to the all-trans ground-state geometry S_0-min. Geometry optimization was performed with the (TD)-B3LYP-D3/cc-pvdz method (see Methods). At the retinal all-*trans* (S_0-min) and 13-*cis* (S_0(10ps)-min) ground-state geometries, the C13=C14 torsion angle equals -162 and -41 degrees, respectively. Initial excited-state relaxation does not result in any significant change of the torsion angle. The bond-length alternation (BLA) is more pronounced, especially for the elongation of the C13=C14 bond, at the CT(W86)-min and CT(Y185)-min as compared to the S_1-min. Furthermore, a significant change in hydrogen bond distances is predicted in the CT states – water molecule W402 moves away from the protonated Schiff base nitrogen atom, whereas the hydrogen bonds formed by Tyr195 and Trp86 are shortened. Overall, relaxation of hydrogen bonds in the CT states is much more pronounced as compared to that in the S_1 state which is consistent with the significantly larger dipole moments of the CT states as compared to the S_1 state (see Supplementary Table 1).

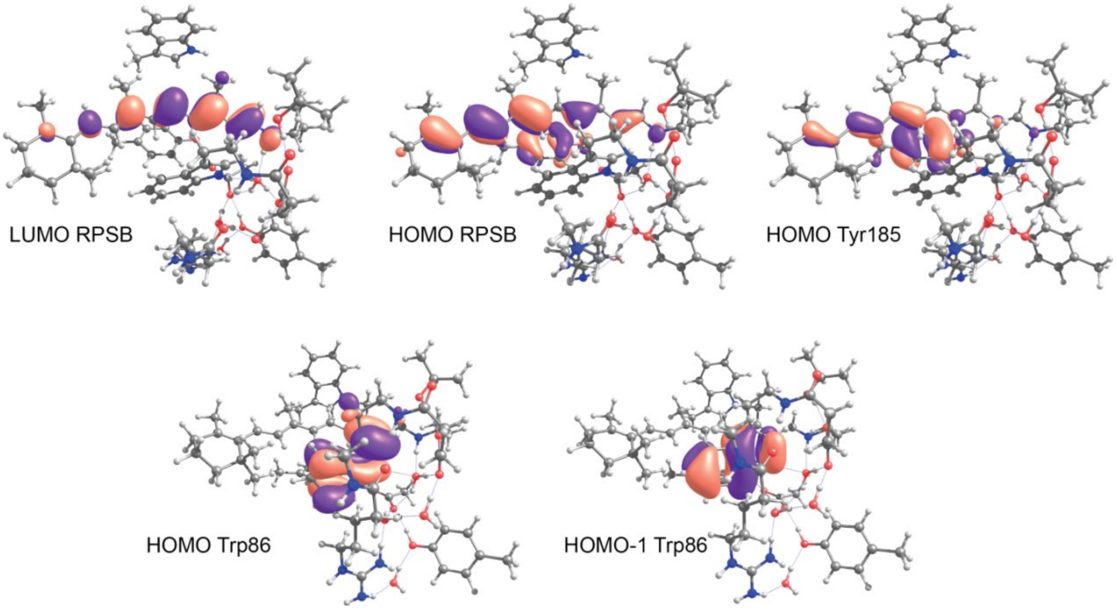

Supplementary Figure 8. CASSCF active space (8,5). For residue labelling, see Fig. 3a (main text).

a

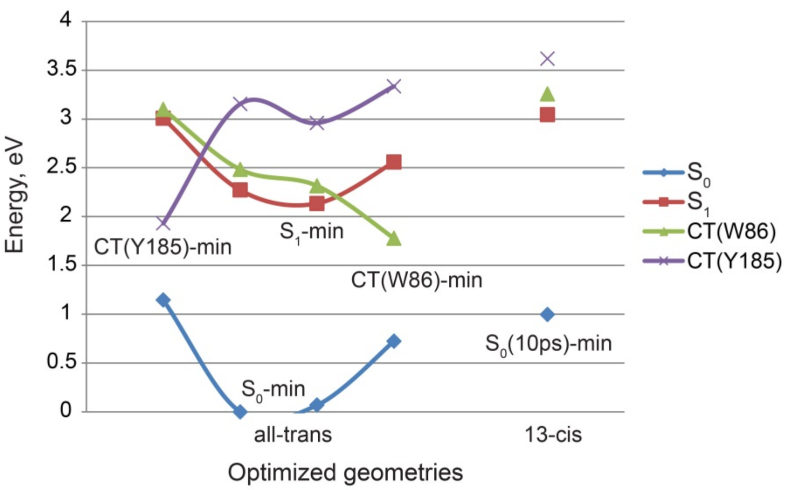

b

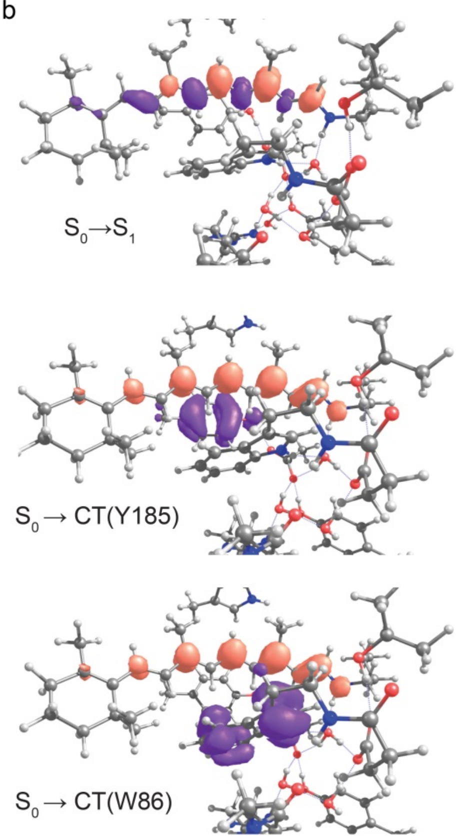

c

Estimated electronic coupling (cm^{-1}).

Coupled states	Excited-state geometry		
	S_1-min	CT(W86)-min	CT(Y185)-min
S_1-CT(W86)	216	223	314
S_1-CT(Y185)	919	1090	805
CT(W86)-CT(Y185)	73	113	261

Supplementary Figure 9. The XMCQDPT2-CASSCF(8,5)-SA5 energies and properties of the low-lying excited states at the optimized geometries computed with the (TD)-B3LYP-D3/cc-pvdz method (see Methods). All computed energies, state dipole moments and transition dipole moments are listed in Supplementary Table 1. **a**. Energy comparison at the optimized geometries. Retinal geometries S_0-min, S_1-min, CT(W86)-min and CT(Y185)-min were optimized in the respective electronic states starting from the coordinates of the light-adapted bR structure containing the all-*trans* retinal. The ground-state 13-*cis* retinal geometry S_0-(10ps)-min) corresponds to the 10-ps experimental structure. At the S_0-min optimized geometry, the retinal S_0-S_1 excitation energy is 2.27 eV, whereas the energies of the charge-transfer (CT) states designated as CT(W86) and CT(Y185) states are 2.48 and 3.15 eV, respectively. The transition dipole moments of the CT states with respect to the S_0 state are rather small, especially in comparison with that of the S_1 state, hence these states are difficult to observe in the bR excitation spectrum. Excited-state geometry optimization found three local energy minima on the first-excited potential energy surface. Comparison of their energies indicated that intermolecular electron-transfer is favourable after the initial S_0-S_1 excitation. Population of the CT states during the evolution of excited bR is predicted to result in a red-shifted stimulated emission, as indicated by the S_0-CT(W86)/CT(Y185) energy gaps at the respective excited-state geometries. Notably, these reduced energy gaps are not due to the twisting of the retinal but due to the relaxation of hydrogen bonds and relative orientation of the residues around the retinal triggered by intermolecular charge transfer. The coherent interconversion of the retinal S_1 and CT states supported by the estimated electronic coupling (**c**) may result in coherent oscillations of the red-shifted stimulated emission signal in bR. At the optimized 13-*cis* retinal structure, the S_0-S_1 energy is 2.05 eV, consistent with the red-shifted absorption of the photoproduct. The S_0(10ps)-min energy is by 1 eV higher than the energy of the initial S_0-min, indicating that almost half of the photon-energy could be saved as thermal energy in the early K intermediate. **b**, The difference of the excited-state electron density with respect to the ground-state density (Excited-state *minus* ground-state) demonstrating the electronic structure of excited states.

Positive and negative densities are shown in orange and purple, respectively. **c**. The magnitudes of the electronic coupling estimated using the Mulliken-Hush scheme (see Methods). The estimated electronic couplings are rather large, indicating that population and coherent interconversion of the three excited states, S_1, CT(Y185) and CT(W86) may occur on the femtosecond timescale. Therefore, population of the CT states may contribute to the dynamics of the bR I intermediate.

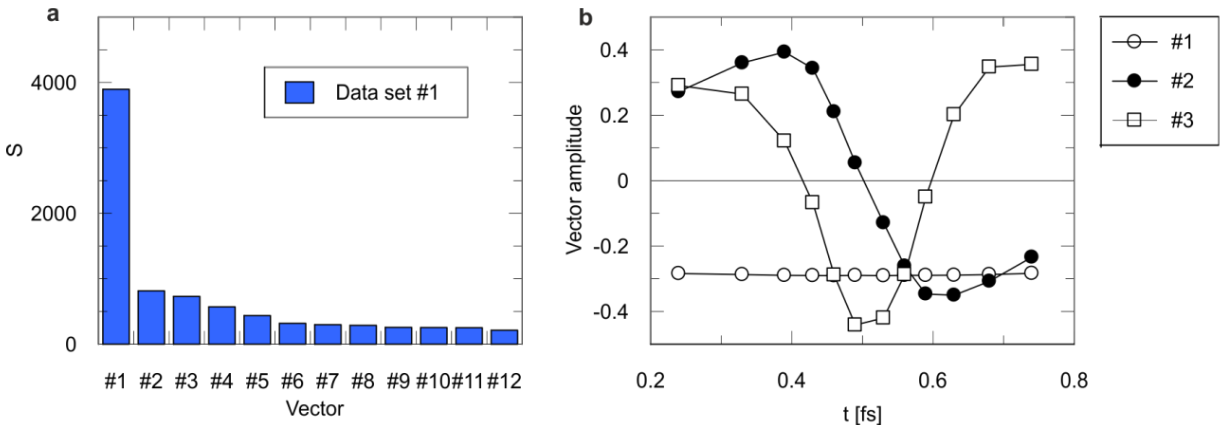

Supplementary Figure 10. Singular value decomposition results. **a**, Weights *S* of the singular vectors. **b**, Time evolution $V_a(t)$ of the first three singular vectors.

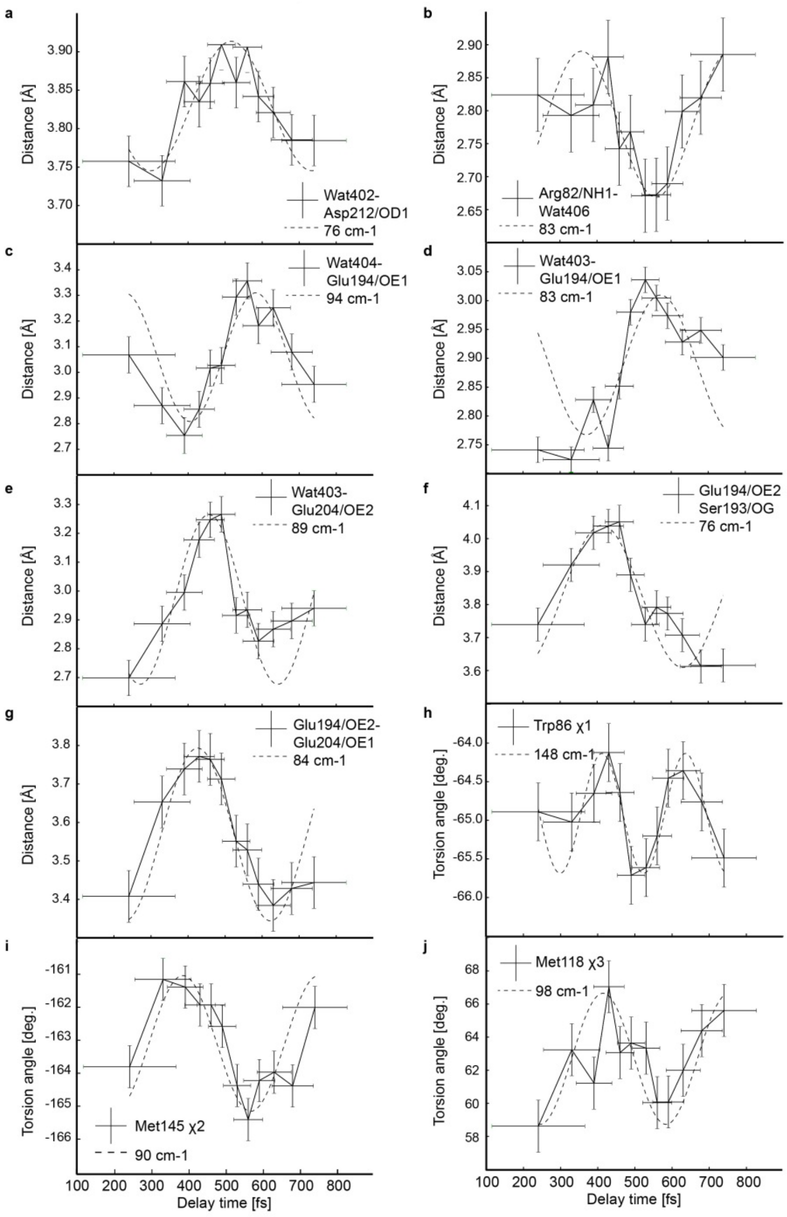

Supplementary Figure 11. Oscillatory modulations in the torsion angles of residues and their distances to other residues and water molecules. The error bars represent standard deviations. The dashed sinusoid shows the main frequency of the oscillation. **a**, The distance between the Asp212 oxygen OD1 and water Wat402 oscillates in time with a period of 76 cm^{-1} similarly to the oxygen OD2-Wat402 distance (80±9 cm^{-1}, Fig 8.e). **b**, The motion is likely propagated via the water network towards Arg82, whose nitrogen NH1 to water Wat406 distance oscillates with 83 cm^{-1}. **c-g**, Similar oscillatory modulation occur in the residues at the proton release site towards the extracellular. This includes in addition to Fig.8f distances between Glu194/OE1 and waters Wat404 (94 cm^{-1}, c) and Wat403 (83 cm^{-1}, d), between Glu204/OE2 and Wat403 (89 cm^{-1}, e) and between Glu194/OE2 and Ser193/OG (76 cm^{-1}, f) and Glu204/OE1 (84 cm^{-1}, g). h-j, Oscillations of torsion angles of residues lining the retinal pocket (in addition to Fig 8.g-i) include Trp86 χ_1 (148 cm^{-1}, h), Met118 χ_3 (98 cm^{-1}, i) and Met145 χ_2 (90 cm^{-1}, j).

The error bars were derived as described in the Supplementary Methods. Source data are provided for Supplementary Figure 11a-j as a Source Data file.

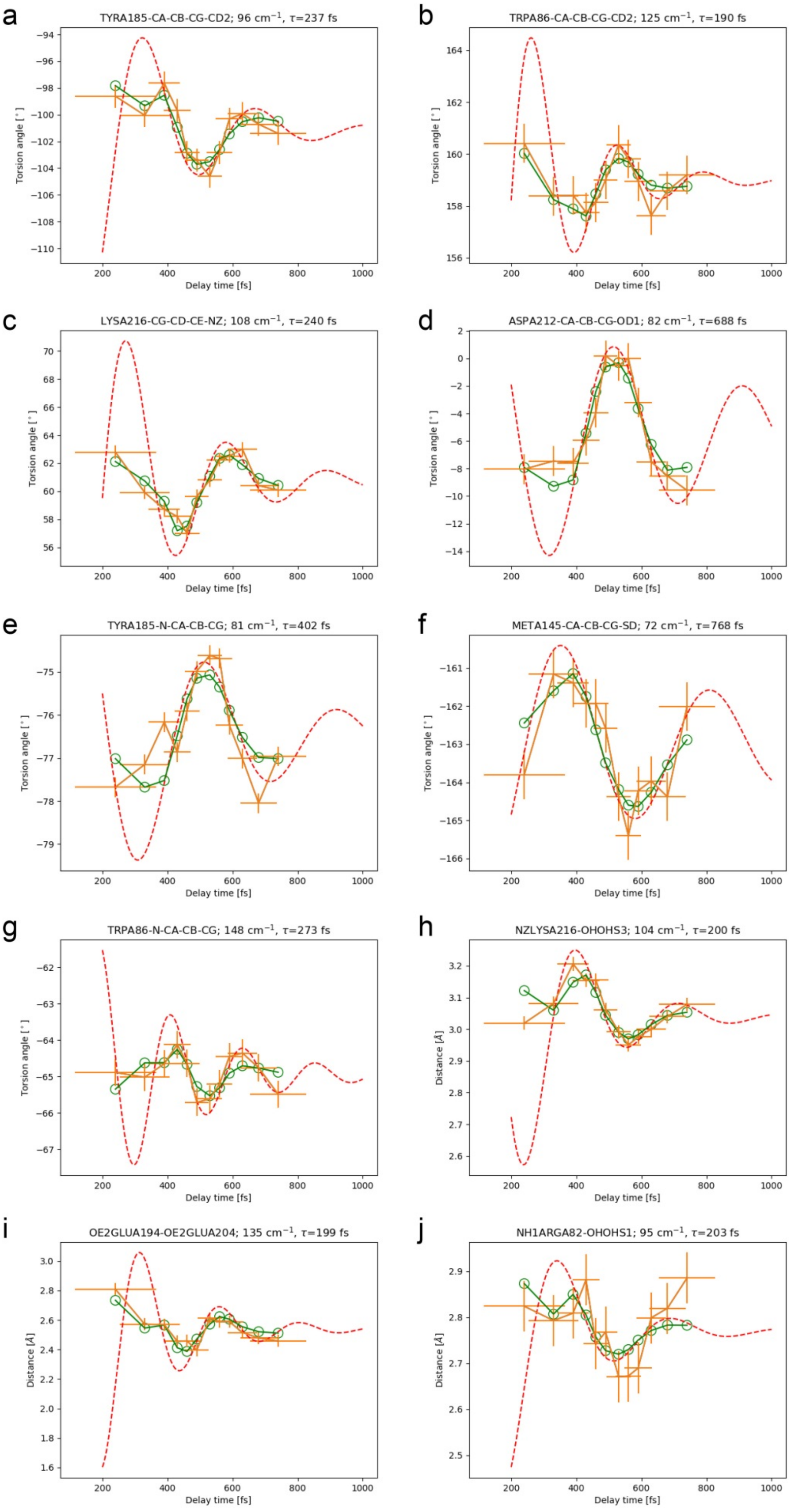

Supplementary Figure 12. Effect of the binning of the crystallographic data on the oscillations. The orange crosses are the measured values from the crystal structures with standard deviations. The dashed red curve is a simulated damped sinus, and the green circles are values that would be obtained

from the red curve if it had been binned in the same manner as the crystallographic data. The parameters of the red curve were obtained by minimizing the squared differences between these binned, simulated values (green) and the actual measured values (orange). Starting parameters for the minimizations included the oscillation periods as obtained from the crystal structures (Fig. 8, Supp. Fig. 11). Oscillations are shown for **a** Tyr185 χ_2 angle, **b** Trp86 χ_2 angle, **c** Lys216 χ_4 angle, **d** Asp212 χ_2 angle, **e** Tyr185 χ_1 angle, **f** Met145 χ_2 angle, **g** Trp86 χ_1 angle, **h** distance Lys216/Nz to Wat402, **i** distance Glu194/OE2 to Glu204/OE2, **j** distance Arg82/NH1 to Wat406.

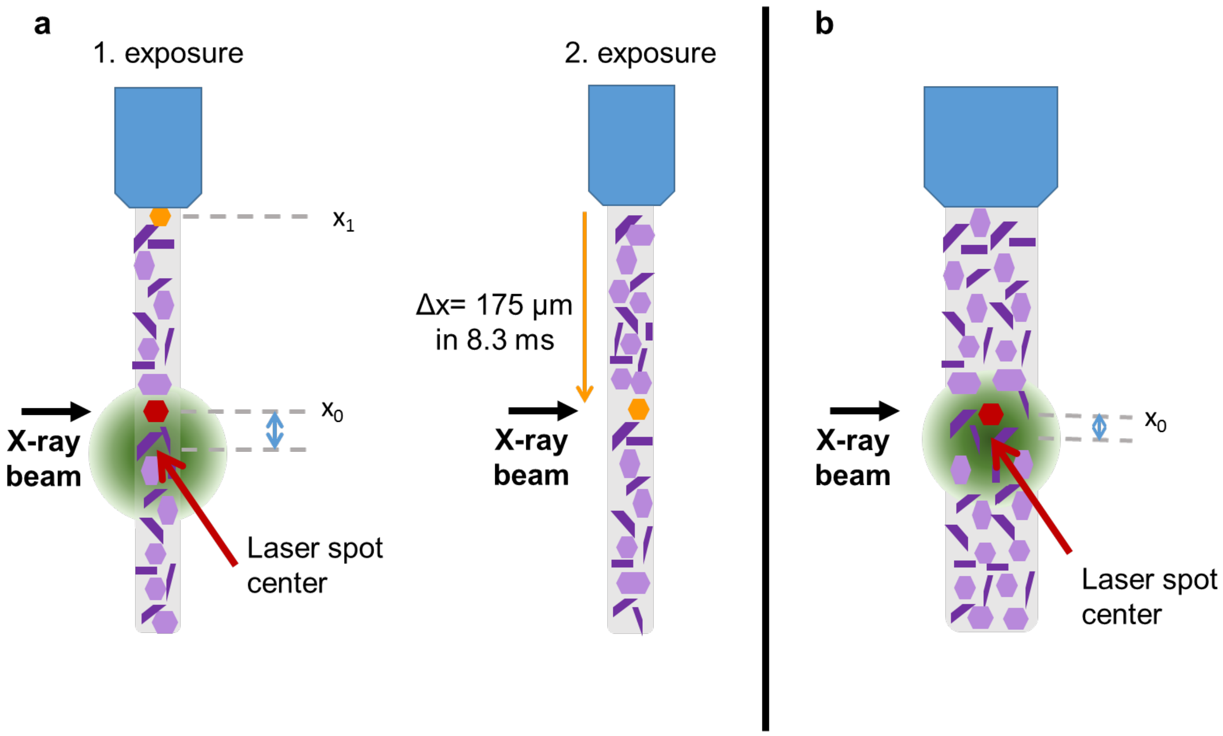

Supplementary Figure 13. Injection and illumination protocol of the Nogly et al.[32] (a) and this (b) experiment. The blue arrows indicate the offset between the optical laser centre and the X-ray interaction region. **a,** Experimental conditions as described in the publication[32]. The 95 µm ($1/e^2$) laser beam is offset by 50 µm downstream of the X-ray interaction point. During the first exposure, the red crystal in the laser spot is probed at position x_0 with an ultrafast time delay, yielding e.g. 16 % occupancy of the 10 ps intermediate. During the second X-ray exposure, 8.3 ms after the first one, the orange crystal is probed and yields the M intermediate with a 10 % occupancy. The distance of the orange crystal at position x_1 to the laser centre is in total 225 µm. It seems physically impossible for a Gaussian-like beam that the photon density is high enough at x_1, the position of the orange crystal, to yield very similar intermediate occupancy as at the red crystal orientation. **b,** in our experiment the 99 µm ($1/e^2$) laser beam centre is offset by 20-30 µm downstream of the X-ray interaction point. The red crystal at position x_0 is probed with an ultrafast time delay.

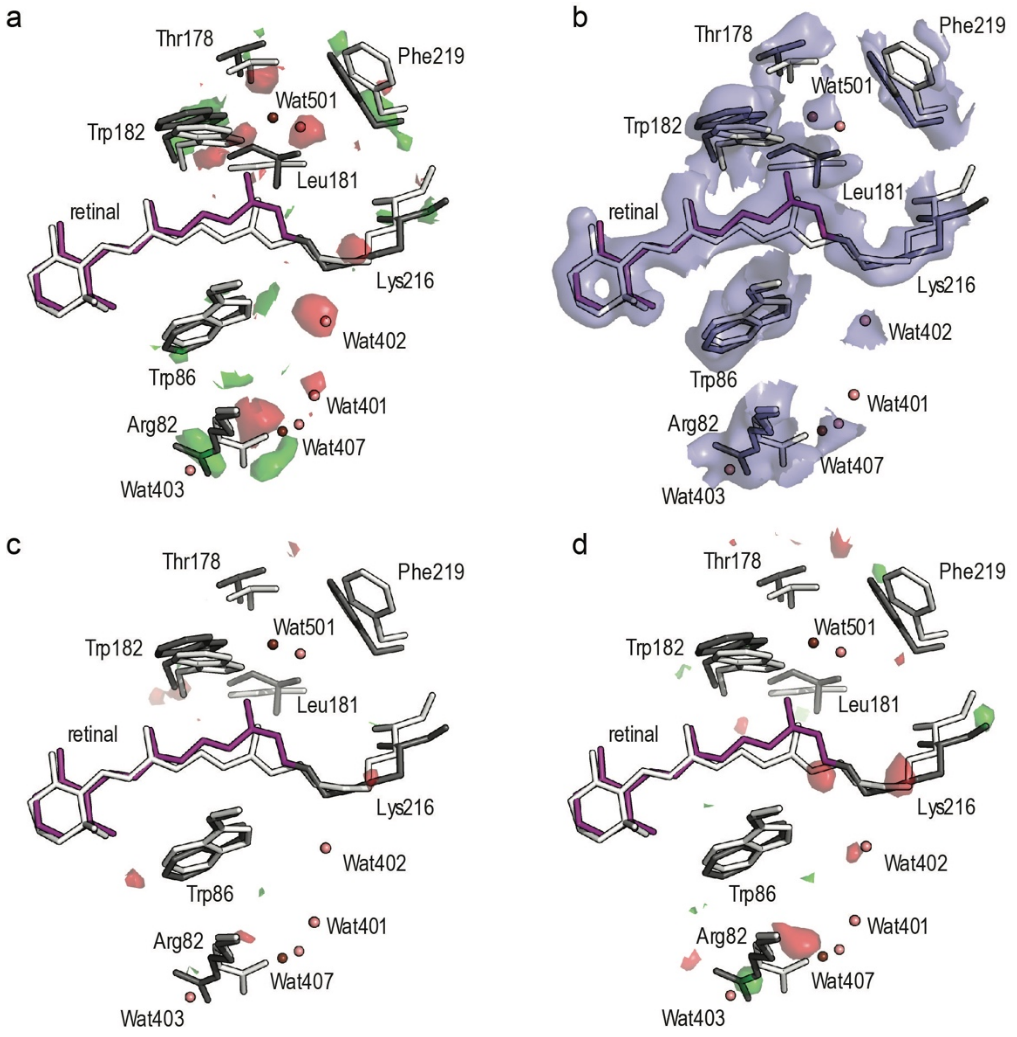

Supplementary Figure 14. The 33 ms time-point structure. The unpumped state retinal and protein residues are shown as white sticks, whereas the pumped state retinal and protein are shown as purple- and grey sticks, respectively **a, b,** Close-up of the retinal and its immediate surrounding in the 33 ms time point structure. In **a**, the q-weighted pumped-unpumped difference electron density map is shown, contoured at +3.0s (green) and -3.0s (red). In **b**, the 15% extrapolated electron density map is shown, contoured at 1.0 s (blue). **c,** F_o-F_o difference electron density map between our 33 ms structure and the 1.725 ms structure (5B6Z[7]) showing good agreement between the structures. **d,** $F_{extrapol}$-$F_{extrapol}$ difference density map between our 33 ms structure and the 8.3 ms structure (6G7L) reported recently[32], showing a larger contribution of light-induced changes in our structure. For **c** and **d**, water molecules from the dark state and pumped structures are shown as salmon and dark red spheres, respectively.

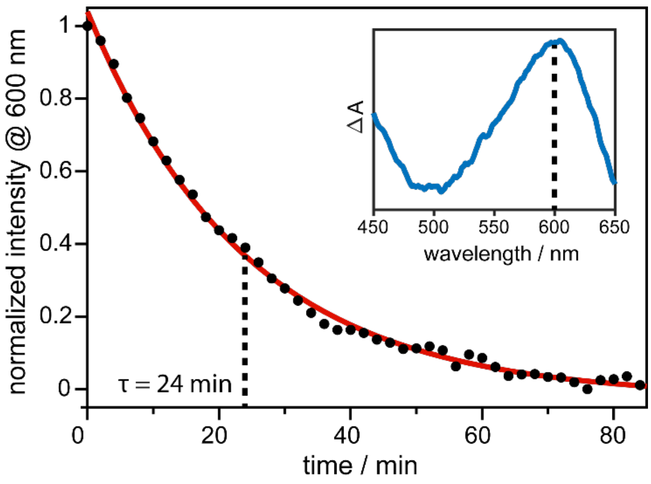

Supplementary Figure 15. Decay of the light-adapted state in bR microcrystals recorded at 600 nm (black dots) where the largest difference in absorption is observed (see inset). The measured absorption difference was normalized to 100% at time 0 and to 0% at 85 min. An exponential function was fitted to the trace, yielding a time constant of 24 min (red continuous line). Inset: the difference absorbance spectrum (blue) between the light and the dark-adapted states shows a redshift in the electronic transition of retinal.

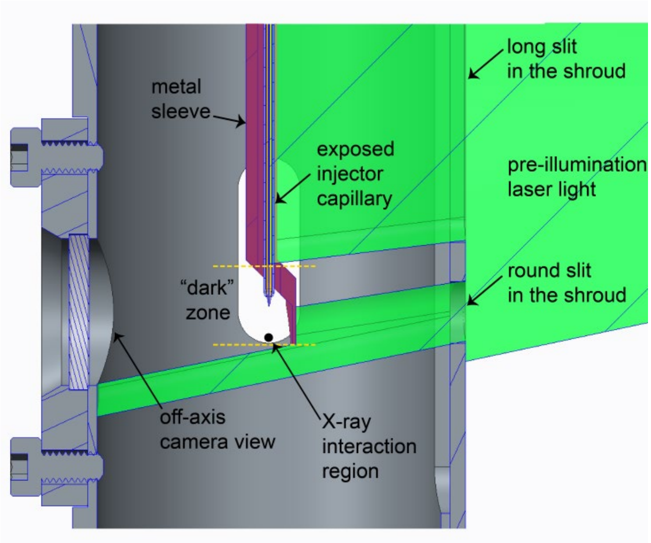

Supplementary Figure 16. Cross-sectional drawing of the injector nozzle in the shroud in the scattering chamber (view along the X-ray and optical pump laser axis). The long upper slit in the shroud allows the pre-illumination light (green) to propagate onto the injector capillary. The lower round slit allows the green light to weakly illuminate the interaction region (with the nozzle moved slightly up) for imaging the sample free-jet with the off-axis camera. The capillary is equipped with a metal sleeve (purple) that allows the green light to enter the capillary but blocks the light in the last 4-5 mm upstream of the interaction region. This short "dark zone" (yellow dashed lines) allows the light-adapted crystals to return to the ground state prior to being pumped with the optical pump laser.

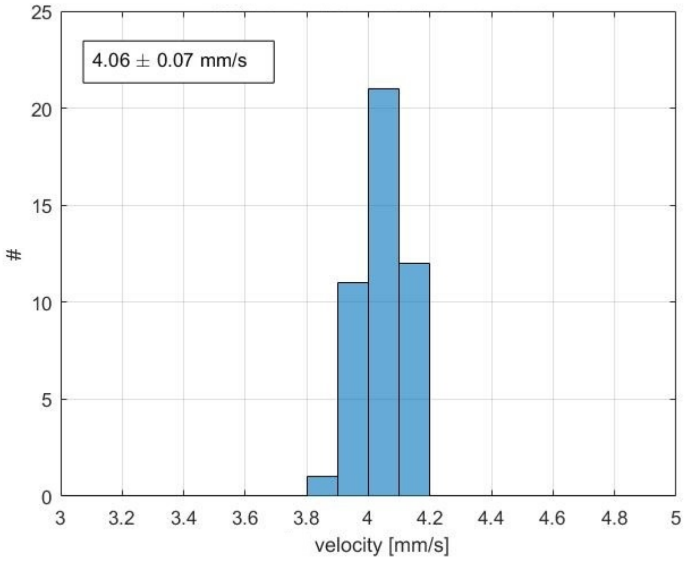

Supplementary Figure 17. Histogram of the stream velocities measured during two consecutive runs from the time-resolved data collection. Based on the jet diameter (100 µm) and the flow rate (1.9 µl/min), the calculated stream velocity is 4 mm/s. The histogram shows that the average measured stream velocity based on tracking features is 4.06 ± 0.07 mm/s, which corresponds closely to the calculated value.

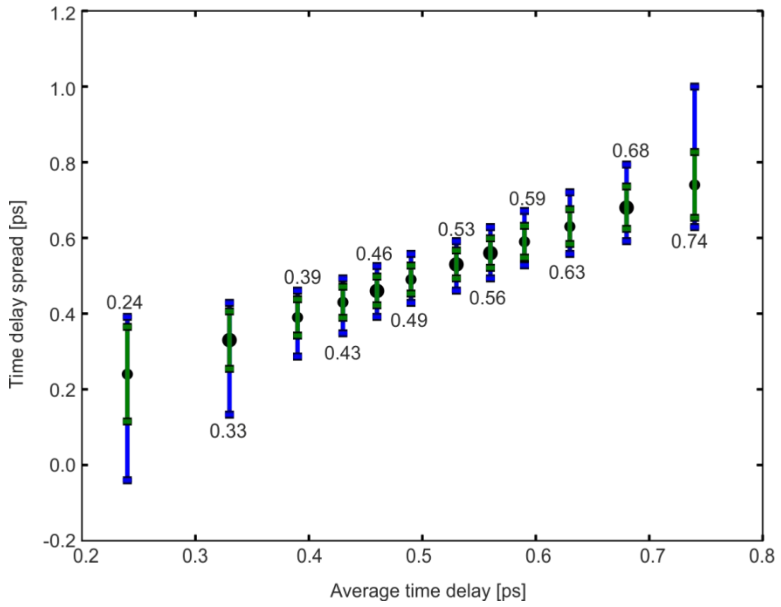

Supplementary Figure 18. Spread in the time delays for the sub-ps delays. The green bars indicate the standard deviation for the distribution of time delays in each dataset, and the blue bars indicate the minimum and maximum time delays. The average time delays are indicated by a black filled circle.

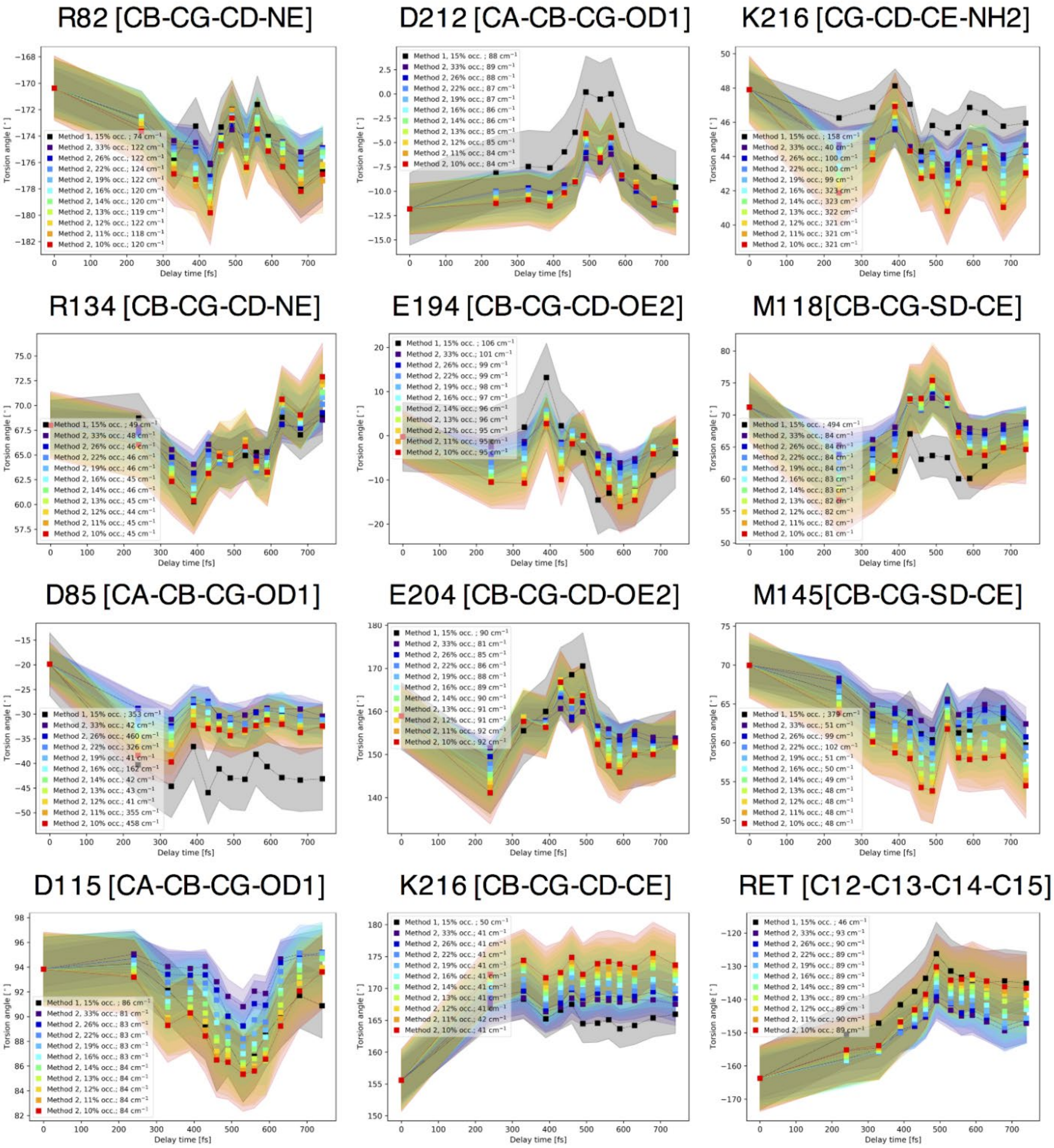
R82 [CB-CG-CD-NE]
D212 [CA-CB-CG-OD1]
K216 [CG-CD-CE-NH2]
R134 [CB-CG-CD-NE]
E194 [CB-CG-CD-OE2]
M118[CB-CG-SD-CE]
D85 [CA-CB-CG-OD1]
E204 [CB-CG-CD-OE2]
M145[CB-CG-SD-CE]
D115 [CA-CB-CG-OD1]
K216 [CB-CG-CD-CE]
RET [C12-C13-C14-C15]
Torsion angle [°]
Delay time [fs]

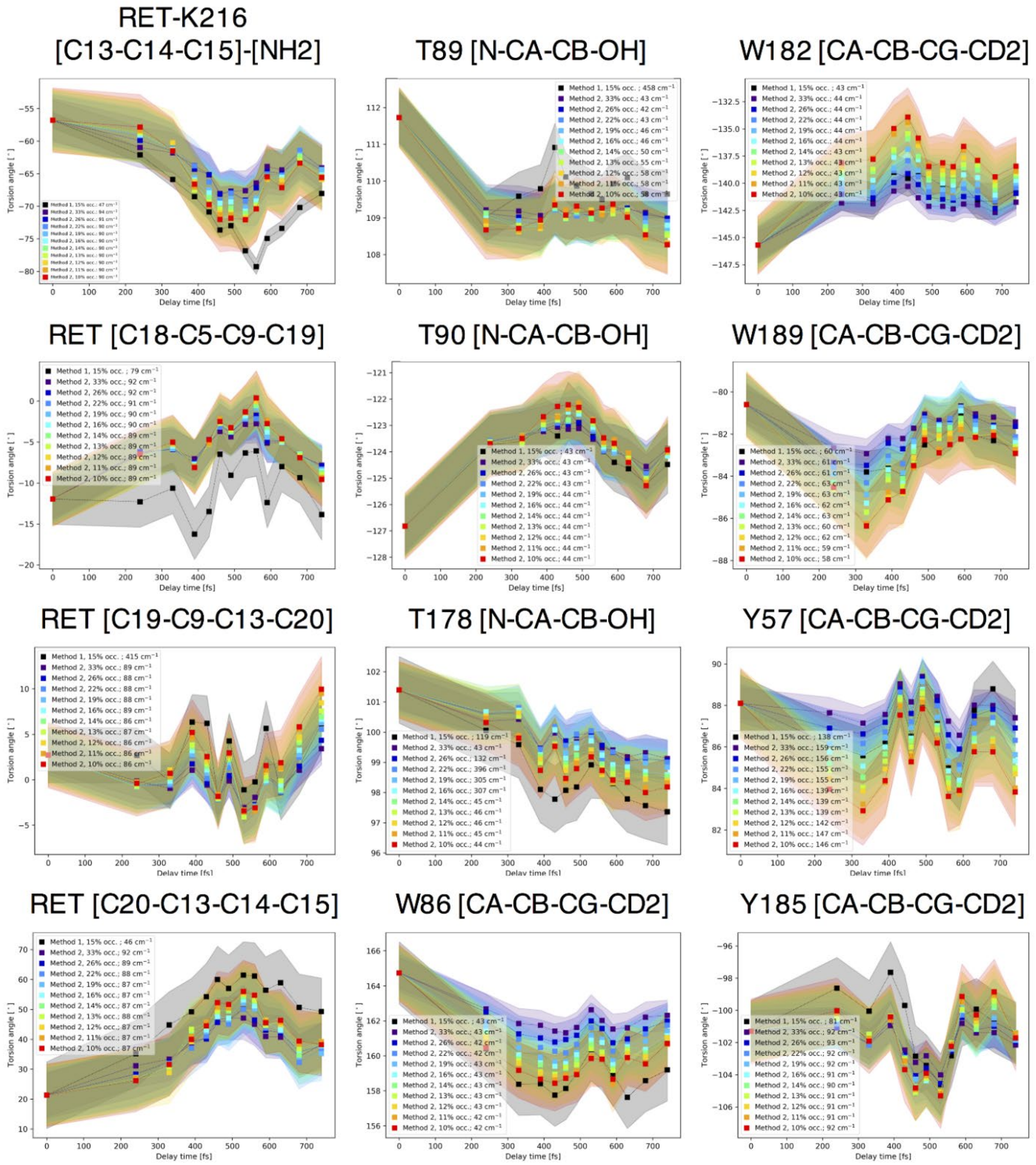

Supplementary Figure 19. Oscillations are visible in the sub-ps time-delay structures, regardless of the software used to generate extrapolated structures factors and maps. Select torsion angles from residues in the close vicinity of the retinal are plotted as a function of the pump-probe delay. Black squares correspond to the sub-ps time-delay structures refined using Method 1, while coloured squares correspond to those refined using Method 2. Corresponding occupancies of the transient structures in the raw datasets (*i.e.*, the inverse of α) and automatically determined oscillation frequencies are indicated.

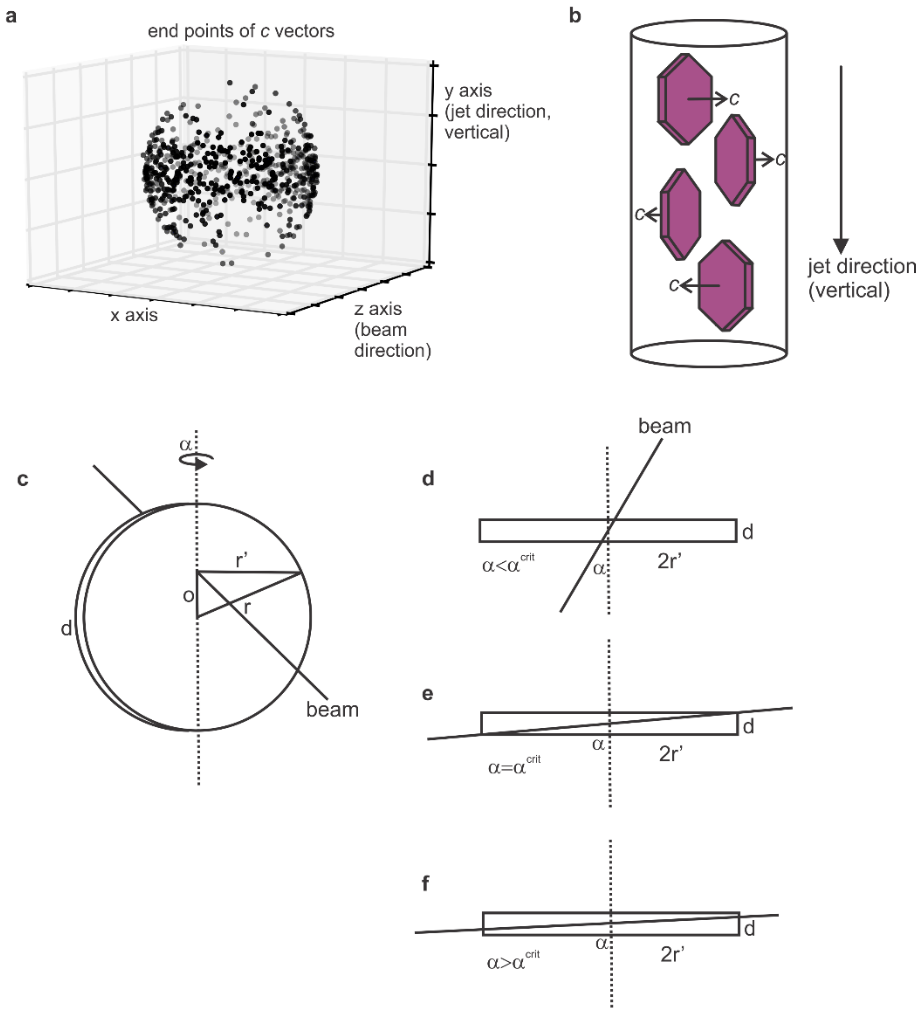

Supplementary Figure 20: Average thickness of the hexagonal plate-shaped bR crystals in the pump beam. Path length through bR crystals in orientations relevant to the experiment. **a**. scatter plot showing the end points of the c-vectors of the crystals contributing to the diffraction data, as retrieved from the CrystFEL output. The indexed crystals are predominantly oriented with their c-axes approximately perpendicular to the direction of the jet as shown in **b**. **c-f**. Formalism for the calculation of the average path length through the crystals, as described in the text.

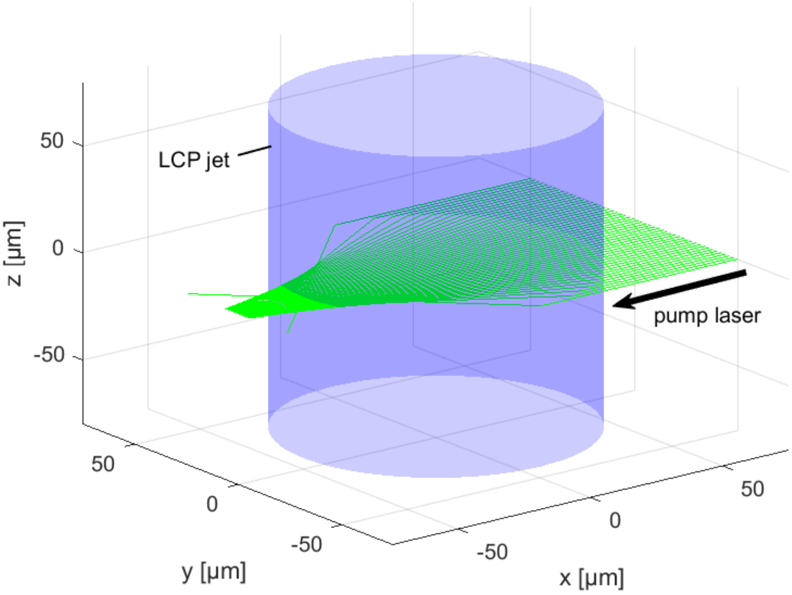

Supplementary Figure 21. **2D Raytracing calculations of a collimated light beam in vacuum impinging on a 100 µm diameter LCP jet.** The cross section of a collimated light beam intersects a cylindrical jet. The fan of rays is refracted at the interface of the jet where the refractive index changes from 1.0 (vacuum) to 1.42 (LCP). For simplicity, refraction was only calculated at the first interface (vacuum to LCP) and omitted for the second interface (LCP to vacuum).

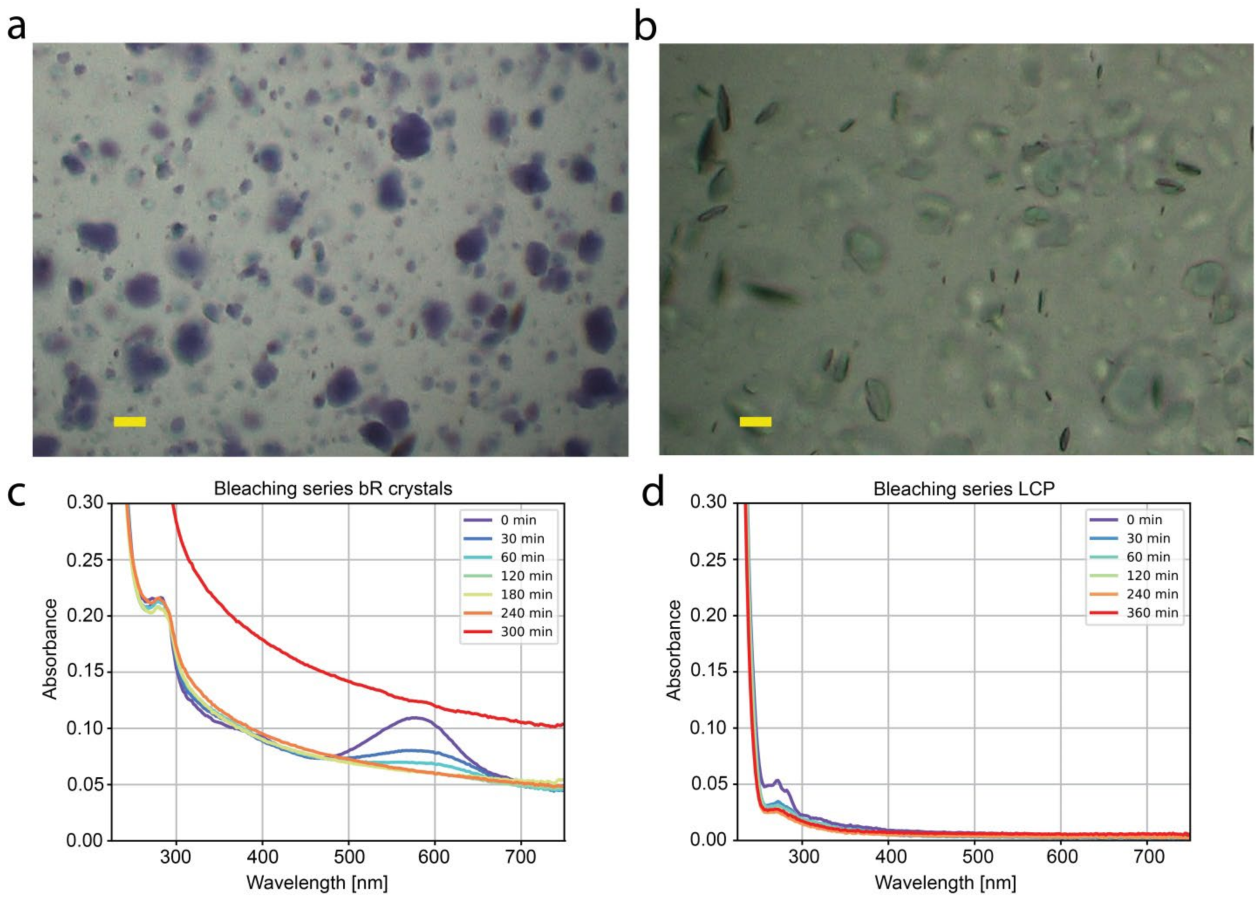

Supplementary Figure 22. Bleaching bR crystals enables measurement of scattering by crystals without the complication of absorption. a, b bR crystals before UV irradiation (**a**) and after 120 min of irradiation (**b**). The yellow bar represents 20 µm. **c, d** Absorption spectra of bR crystals in LCP (**c**) and LCP only (**d**) after bleaching under UV light for 0, 30, 60, 120, 180, 240 and 300 min.

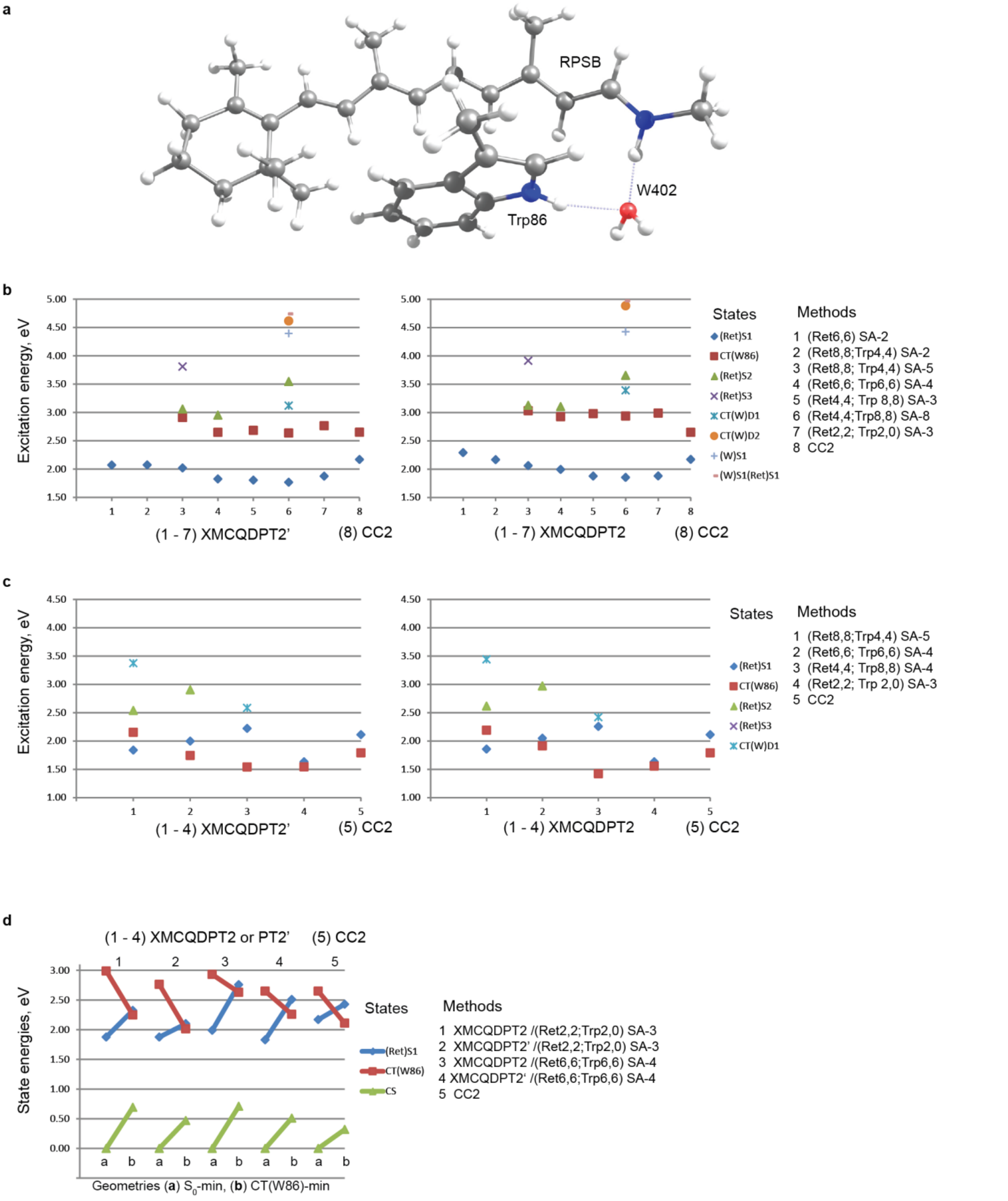

Supplementary Figure 23. Effect of the active-space selection on the CT(W86) energy. The analysed data are collected in Supplementary Tables 8 and 9. **a** Small cluster RPSB-Trp86-W402. **b.** Comparison of the excitation energies computed for the small cluster (**a**) at the S_0-min geometry. **c.** Comparison of the excitation energies computed for the small cluster (**a**) at the CT(W86)-min geometry. **d.** Effect of the computational method on the energies of the (Ret)S_1/CT(W86) state crossing.

SUPPLEMENTARY TABLES

Supplementary Table 1. The XMCQDPT2-CASSCF(8,5)-SA5 energies and properties of the low-lying excited states at the (TD)-B3LYP-D3/cc-pvdz optimized geometries.

State Sx	Assignment	Energy, au		Dipole moment, Debye				Radiative dipole moment, au			
		XMCQDPT2	XMCQDPT2'	x	y	z	norm	Sx-S2	Sx-S3	Sx-S4	Sx-S5
**Geometry S_0-min**											
1	CS	-4253.859171	-4253.857963	5.93	4.83	-9.04	11.84	5.114	0.724	0.957	0.378
2	(Ret)S_1	-4253.775683	-4253.773962	5.35	11.15	-14.07	18.73	-	0.970	1.323	0.621
3	CT(W86)	-4253.767905	-4253.767825	-1.13	-0.85	-24.08	24.12	0.970	-	0.170	0.601
4	CT(Y185)	-4253.743247	-4253.742938	21.85	11.98	-18.13	30.82	1.323	0.170	-	1.302
5	CT(W)D_1	-4253.739245	-4253.739165	1.80	0.80	-26.36	26.43	0.621	0.601	1.302	-
**Geometry S_1-min**											
1	CS	-4253.856646	-4253.855330	5.98	6.27	-9.24	12.66	5.417	0.749	0.928	0.335
2	(Ret)S_1	-4253.780708	-4253.778986	5.46	10.67	-13.81	18.29	-	0.960	1.261	0.530
3	CT(W86)	-4253.774076	-4253.773998	-0.95	-1.14	-23.93	23.97	0.960	-	0.150	0.598
4	CT(Y185)	-4253.750423	-4253.750128	22.36	11.62	-17.82	30.86	1.261	0.150	-	1.320
5	CT(W)D_1	-4253.745917	-4253.745847	2.03	0.46	-26.28	26.36	0.530	0.598	1.320	-
**Geometry CT(W86)-min**											
1	CS	-4253.832573	-4253.830806	6.79	8.75	-8.37	13.88	0.208	5.067	0.869	0.856
2	CT(W86)	-4253.793820	-4253.793810	-0.98	-1.17	-22.49	22.54	-	0.242	0.265	0.118
3	(Ret)S_1	-4253.765140	-4253.763419	6.31	11.17	-12.83	18.14	0.242	-	1.089	1.276
4	CT(W)D_1	-4253.744671	-4253.744595	2.88	1.13	-24.95	25.14	0.265	1.089	-	0.470
5	CT(Y185)	-4253.736624	-4253.736481	23.21	12.34	-16.99	31.30	0.118	1.276	0.470	-
**Geometry CT(Y185)-min**											
1	CS	-4253.833675	-4253.832005	3.59	5.37	-10.41	12.25	2.711	4.320	1.494	0.469
2	CT(Y185)	-4253.770047	-4253.769502	16.76	8.63	-17.63	25.82	-	3.260	0.751	0.347
3	(Ret)S_1	-4253.760195	-4253.759069	6.66	7.60	-16.68	19.50	3.260	-	2.070	0.486
4	CT(W86)	-4253.758108	-4253.757767	-2.31	-2.52	-24.59	24.82	0.751	2.070	-	0.587
5	CT(W)D_1	-4253.730143	-4253.730105	-0.30	-2.05	-27.74	27.81	0.347	0.486	0.587	-

Supplementary Table 2: The XMCQDPT2-CASSCF(Ret4,4; Tyr8,8)-SA20 energies and properties computed at the (TD)-B3LYP-D3/cc-pvdz optimized geometries.

State Sx	Assignment	Energy, au		Relative energy, eV	Dipole moment, Debye			
		XMCQDPT2	XMCQDPT2'		x	y	z	norm
**Geometry S_0-min**								
1	CS	-4254.05433	-4253.877345	0.00	7.02	2.15	-9.84	12.27
2	(Ret)S_1	-4253.952127	-4253.795069	2.24	6.00	9.66	-12.66	17.02
3	CT(W86)	-4253.924533	-4253.783408	2.56	-0.54	-1.27	-24.83	24.87
4	CT(W)D_1	-4253.904923	-4253.76209	3.14	2.18	1.27	-25.54	25.67
5	(Ret)S_2	-4253.899699	-4253.753096	3.38	6.13	7.13	-13.66	16.59
6	(W)S_1	-4253.891873	-4253.721503	4.24	6.07	5.77	-10.56	13.48
7	(Ret)S_3	-4253.869226	-4253.71946	4.30	5.26	11.40	-14.58	19.24
8	(W)S_2	-4253.862723	-4253.714248	4.44	4.49	2.56	-7.54	9.14
9	CT(Ret)D_1	-4253.85058	-4253.713683	4.45	3.47	3.78	-18.69	19.38
10	CT(W)D_2	-4253.848527	-4253.708566	4.59	3.26	3.27	-19.55	20.09
11	(Ret)S_4	-4253.83581	-4253.698829	4.86	1.40	-0.45	-20.63	20.68
12	(W)S_3	-4253.834432	-4253.693287	5.01	3.69	2.61	-13.15	13.90
13	(W)S_1(Ret)S_1	-4253.818827	-4253.676745	5.46	5.07	4.53	-17.39	18.67
14	CT(Ret)D_2	-4253.816202	-4253.66662	5.73	3.75	2.54	-21.51	21.98
15	(W)S_4	-4253.809077	-4253.658313	5.96	6.87	5.54	-10.81	13.95
16	(W)S_2(Ret)S_1	-4253.803149	-4253.648458	6.23	6.06	7.62	-11.99	15.44
17	(W)S_5	-4253.781653	-4253.647696	6.25	6.67	5.79	-11.51	14.50
18	(W)S_1(Ret)S_2	-4253.779239	-4253.641772	6.41	6.21	8.33	-12.54	16.28
19	CT(W)D_3	-4253.775683	-4253.628821	6.76	1.81	0.51	-26.44	26.51
20	(W)S_2(Ret)S_2	-4253.753281	-4253.624122	6.89	5.86	6.61	-10.97	14.09
**Geometry S_1-min**								
1	CS	-4254.051066	-4253.877779	-2.04	7.39	1.95	-9.42	12.13
2	(Ret)S_1	-4253.959178	-4253.80289	0.00	6.06	10.80	-12.68	17.72
3	CT(W86)	-4253.93142	-4253.790129	0.35	-0.38	-0.10	-24.87	24.88
4	CT(W)D_1	-4253.913288	-4253.769752	0.90	3.94	3.99	-20.91	21.65
5	(Ret)S_2	-4253.910074	-4253.765577	1.02	4.81	5.08	-18.16	19.46
6	(W)S_1	-4253.887504	-4253.729505	2.00	6.33	6.56	-10.31	13.76
7	(Ret)S_3	-4253.877867	-4253.728125	2.03	5.45	12.22	-14.62	19.82
8	(W)S_2	-4253.857761	-4253.722367	2.19	4.54	3.20	-8.01	9.75
9	CT(W)D_2	-4253.855027	-4253.715071	2.39	2.21	1.57	-22.23	22.40
10	CT(Ret)D_1	-4253.854	-4253.714171	2.41	2.17	-1.11	-22.27	22.40
11	(Ret)S_4	-4253.851985	-4253.707464	2.60	3.75	3.94	-17.61	18.44
12	(W)S_1(Ret)S_1	-4253.831815	-4253.705083	2.66	2.43	-0.28	-26.82	26.93
13	(W)S_3	-4253.828035	-4253.694125	2.96	5.72	5.42	-9.28	12.18
14	(W)S_2(Ret)S_1	-4253.821657	-4253.683339	3.25	6.73	7.97	-12.17	16.02
15	CT(W)D_x(Ret)D_y	-4253.80989	-4253.664761	3.76	0.11	0.79	-24.37	24.39
16	(W)S_1(Ret)S_2	-4253.808216	-4253.663386	3.80	6.34	7.95	-11.72	15.52
17	(W)S_4	-4253.803816	-4253.654128	4.05	7.04	6.23	-11.05	14.51
18	(W)S_2(Ret)S_2	-4253.783614	-4253.647857	4.22	5.14	6.39	-16.51	18.43
19	CT(W)D_3	-4253.782851	-4253.647403	4.23	3.14	2.95	-22.73	23.13

20	(W)S_3(Ret)S_1	-4253.764189	-4253.639541	4.44	6.17	7.16	-10.76	14.32
**Geometry CT(W86)-min**								
1	CS	-4254.027012	-4253.856776	-1.31	8.57	4.40	-8.57	12.90
2	CT(W86)	-4253.952068	-4253.808508	0.00	0.25	-0.28	-23.07	23.07
3	(Ret)S_1	-4253.938901	-4253.788948	0.53	5.95	16.78	-12.64	21.83
4	CT(W)D_1	-4253.904936	-4253.763809	1.22	2.68	2.34	-25.57	25.82
5	(Ret)S_2	-4253.885451	-4253.746094	1.70	7.98	7.33	-11.33	15.68
6	CT(Ret)D_1	-4253.873849	-4253.744045	1.75	1.74	-5.63	-23.14	23.88
7	CT(W)D_2	-4253.864345	-4253.724403	2.29	2.12	1.77	-25.10	25.25
8	(W)S_1	-4253.860431	-4253.722267	2.35	7.39	6.50	-8.46	12.98
9	(W)S_2	-4253.851933	-4253.708243	2.73	5.68	5.34	-7.04	10.51
10	(W)S_1(Ret)S_1	-4253.842858	-4253.700364	2.94	7.34	7.54	-10.37	14.77
11	CT(W)D_2(Ret)D_1	-4253.824311	-4253.699136	2.98	2.46	-0.17	-24.94	25.06
12	CT(W)D_x(Ret)D_y	-4253.821295	-4253.694611	3.10	1.15	0.92	-24.11	24.15
13	CT(W)D_3	-4253.801619	-4253.670539	3.75	0.34	1.16	-23.03	23.06
14	(W)S_1(Ret)S_2	-4253.797707	-4253.665391	3.89	7.46	7.20	-10.84	15.00
15	(W)S_3	-4253.79748	-4253.65947	4.06	7.03	8.21	-9.75	14.56
16	CT(W)D_x(Ret)D_y	-4253.783385	-4253.653058	4.23	2.53	-1.31	-24.72	24.88
17	(W)S_x(Ret)Sy	-4253.781848	-4253.64666	4.40	5.86	4.84	-16.03	17.74
18	CT(W)D_4	-4253.78102	-4253.638473	4.63	4.40	3.59	-19.52	20.33
19	(W)S_x(Ret)S_y	-4253.769477	-4253.635699	4.70	8.03	8.31	-11.16	16.06
20	(W)S_x(Ret)S_y	-4253.748621	-4253.629396	4.87	6.88	8.02	-10.01	14.55

Supplementary Table 3: The XMCQDPT2-CASSCF(Ret4,4; Tyr8,8)-SA20 transition dipole moments (au) computed at the (TD)-B3LYP-D3/cc-pvdz optimized geometries. Assignment of the states is given in Supplementary Table 2.

**Geometry S_0-min**																			
States	1	2	3	4	5	6	7	8	9	10	11	12	13	14	15	16	17	18	19
2	4.483																		
3	0.081	0.162																	
4	0.448	0.340	0.270																
5	0.164	1.741	0.111	1.949															
6	0.328	0.405	0.145	0.556	0.171														
7	0.513	4.956	0.153	0.274	0.457	0.199													
8	0.718	0.927	0.172	0.105	0.127	0.367	0.664												
9	0.041	0.065	0.804	0.114	0.043	0.068	0.071	0.188											
10	0.033	0.033	0.797	0.093	0.038	0.054	0.066	0.170	3.358										
11	0.556	0.147	1.429	0.036	0.059	0.158	0.135	0.150	0.489	0.479									
12	0.900	0.176	0.959	0.138	0.032	0.227	0.120	0.233	0.182	0.406	3.096								
13	0.037	0.168	0.084	0.983	0.503	0.079	0.270	0.036	0.189	0.206	0.234	0.212							
14	0.063	0.207	0.063	1.131	0.554	0.096	0.368	0.044	0.174	0.110	0.305	0.113	3.259						
15	0.489	0.069	0.093	0.049	0.055	0.091	0.107	0.499	0.115	0.128	0.222	0.329	0.250	0.315					
16	0.012	0.015	0.034	0.177	0.033	0.017	0.044	0.010	0.104	0.125	0.093	0.060	0.412	0.531	0.042				
17	0.581	0.083	0.074	0.049	0.053	0.122	0.075	0.112	0.104	0.114	0.119	0.147	0.117	0.109	0.379	0.020			
18	0.072	0.013	0.118	0.036	0.004	0.012	0.011	0.018	0.237	0.246	0.195	0.117	0.098	0.060	0.049	0.081	0.179		
19	0.095	0.064	0.148	0.271	0.115	0.032	0.058	0.069	0.366	0.341	0.049	0.097	0.150	0.157	0.105	0.194	1.225	0.555	
20	0.007	0.008	0.189	0.036	0.005	0.003	0.010	0.012	0.061	0.068	0.070	0.036	0.034	0.025	0.013	0.020	0.027	0.026	0.074
**Geometry S_1-min**																			
States	1	2	3	4	5	6	7	8	9	10	11	12	13	14	15	16	17	18	19
2	4.696																		
3	0.089	0.180																	
4	0.467	0.931	0.150																

5	0.097	1.765	0.159	3.312															
6	0.234	0.270	0.094	0.195	0.155														
7	0.621	5.369	0.039	0.287	0.285	0.147													
8	0.708	0.527	0.317	0.072	0.123	0.396	0.250												
9	0.178	0.044	0.986	0.138	0.088	0.132	0.097	1.406											
10	0.066	0.130	2.439	0.352	0.280	0.057	0.106	0.082	1.496										
11	0.070	0.048	1.854	0.315	0.250	0.038	0.103	0.704	2.201	2.668									
12	0.075	0.272	0.402	1.393	1.342	0.059	0.473	0.018	0.043	0.297	0.248								
13	0.778	0.231	0.144	0.068	0.057	0.321	0.116	0.205	0.147	0.047	0.065	0.338							
14	0.021	0.022	0.084	0.204	0.137	0.009	0.048	0.052	0.194	0.113	0.199	0.675	0.031						
15	0.149	0.118	2.500	0.546	0.363	0.081	0.041	0.215	0.109	0.516	0.168	0.191	0.388	0.139					
16	0.009	0.007	0.044	0.110	0.072	0.007	0.019	0.025	0.073	0.086	0.120	0.324	0.029	0.181	0.072				
17	0.416	0.025	0.444	0.082	0.104	0.147	0.060	0.421	0.141	0.098	0.069	0.067	0.453	0.027	1.085	0.019			
18	0.043	0.023	0.143	0.124	0.116	0.011	0.024	0.077	0.341	0.184	0.091	0.022	0.027	0.110	0.101	0.123	0.025		
19	0.071	0.050	0.141	0.130	0.144	0.013	0.037	0.102	0.366	0.203	0.212	0.009	0.056	0.167	0.075	0.137	0.018	3.087	
20	0.007	0.023	0.107	0.095	0.075	0.004	0.023	0.006	0.036	0.083	0.072	0.027	0.006	0.011	0.095	0.017	0.017	0.060	0.078
**Geometry CT(W86)-min**																			
States	1	2	3	4	5	6	7	8	9	10	11	12	13	14	15	16	17	18	19
2	0.289																		
3	4.022	0.542																	
4	0.455	0.225	0.699																
5	0.533	0.298	0.722	0.453															
6	0.030	3.422	0.165	0.253	0.569														
7	0.029	0.990	0.082	0.192	0.096	0.146													
8	0.473	0.097	0.234	0.097	0.056	0.023	0.455												
9	1.008	0.238	0.341	0.033	0.092	0.108	0.351	0.953											
10	0.008	0.147	0.024	0.100	0.010	0.133	0.197	0.014	0.012										
11	0.154	1.198	0.575	1.909	0.489	0.660	0.189	0.046	0.220	0.086									
12	0.111	1.918	0.216	0.864	0.306	0.706	0.173	0.105	0.260	0.160	0.768								

13	0.049	0.778	0.064	0.261	0.024	0.119	0.572	0.049	0.107	0.214	0.093	0.045							
14	0.182	0.209	0.074	0.105	0.024	0.044	0.157	0.036	0.151	0.149	0.210	0.165	1.190						
15	0.563	0.140	0.186	0.040	0.036	0.019	0.109	0.132	0.495	0.051	0.091	0.032	0.739	0.275					
16	0.042	0.220	0.078	0.027	0.079	0.769	2.054	0.114	0.105	0.108	0.364	0.183	0.312	0.082	0.036				
17	0.049	0.217	0.044	0.121	0.033	0.183	0.560	0.046	0.040	0.216	0.182	0.093	0.139	0.270	0.079	0.425			
18	0.061	0.217	0.063	0.152	0.037	0.174	0.553	0.058	0.036	0.233	0.116	0.107	0.183	0.215	0.077	0.337	3.231		
19	0.005	0.083	0.007	0.069	0.006	0.080	0.135	0.015	0.007	0.122	0.076	0.059	0.081	0.139	0.040	0.346	0.132	0.175	
20	0.033	0.123	0.148	0.137	0.035	0.082	0.063	0.017	0.015	0.451	0.177	0.132	0.044	0.017	0.011	0.065	0.102	0.108	0.033

Supplementary Table 4. Crystallographic Data and Refinement Statistics

	dark, cell 1	dark, cell 2	33 ms		dark unrestrained*	1 ps	3 ps	10 ps
**Data collection**								
Space group	$P6_3$	$P6_3$	$P6_3$		$P6_3$	$P6_3$	$P6_3$	$P6_3$
No. indexed images	22,485	69,518	9,022		10,000	11,590	9,739	8,997
Cell dimensions (Å)								
a, b, c (Å)	61.7, 61.7, 109.2	62.1, 62.1, 110.5	62.1, 62.1, 109.6		62.1, 62.1, 110.5	61.7, 61.7, 109.3	62.0 62.0, 110.3	61.7, 61.7, 109.4
α, β, γ (°)	90, 90, 120	90, 90, 120	90, 90, 120		90, 90, 120	90, 90, 120	90, 90, 120	90, 90, 120
Resolution (Å)	20-1.7	20-1.8	20-2.1		20-1.8	20-1.8	20-1.9	20-1.8
	(1.8-1.7)	(1.9-1.8)	(2.2-2.1)		(1.9-1.8)	(1.9-1.8)	(2.0-1.9)	(1.9-1.8)
R_{split}	0.069	0.057	0.143		0.154	0.108	0.124	0.119
	(0.543)	(0.623)	(0.714)		(1.947)	(0.793)	(0.751)	(0.607)
CC*	0.999	0.999	0.995		0.996	0.997	0.996	0.996
	(0.920)	(0.893)	(0.862)		(0.458)	(0.845)	(0.854)	(0.901)
$I / \sigma I$	9.9 (2.0)	11.6 (1.8)	5.3 (1.5)		4.3 (0.6)	6.8 (1.4)	5.9 (1.4)	6.4 (1.7)
Completeness (%)	100 (100)	100 (100)	100 (100)		100 (100)	100 (100)	100 (100)	100 (100)
Redundancy	476 (380)	795 (237)	144 (92)		106 (77)	231 (165)	141 (90)	187 (134)
Estim. twinning fraction (ML)	0.032	0.054	0.066		0.047	0.068	0.046	0.074
**Refinement**				**Real-space refinement**				
Resolution (Å)	20-1.7	20-1.8	20-2.1	Map resolution (Å)	20-1.8	20-1.8	20-1.9	20-1.8
No. reflections	25882	22376	13984	f	n.a.	0.15	0.10	0.10
R_{work} / R_{free}	0.186/0.214	0.179/0.216	0.192/0.244	Model/map fit (CC_{mask})	0.9286	0.8408		0.7785
No. atoms				No. atoms				
Protein	1786	1786	2024	Protein	1786	1786	1786	1786
Ligand/ion	21 (retinal)	21 (retinal)	42 (retinal A, B)	Ligand/ion	21 (retinal)	21 (retinal)	21 (retinal)	21 (retinal)
	206 (lipids)	206 (lipids)	206					
Water	43	38	39	Water	36	36	36	36
B-factors (Å^2)								
Protein	28.7	33.1	36.3					
Ligand/ion	19.4 (retinal)	23.6 (retinal)	24.5, 24.5 (retinal A,B)					
	47.0 (lipids)	50.3 (lipids)	52.9 (lipids)					
Water	39.7	45.0	46.3					
R.m.s. deviations				R.m.s. deviations				
Bond lengths (Å)	0.006	0.007	0.007	Bond lengths (Å)	0.006	0.005		0.005
Bond angles (°)	1.082	1.047	1.121	Bond angles (°)	0.908	0.901		0.792

* the “dark unrestrained” structure was refined in exactly the same way and against data from the same number of images as the sub-ps time delay structures and was used to compare torsional angles in the retinal before and after illumination.

	0.24 ps	0.33 ps	0.39 ps	0.43 ps	0.46 ps	0.49 ps
**Data collection**						
Space group	$P6_3$	$P6_3$	$P6_3$	$P6_3$	$P6_3$	$P6_3$
No. indexed images	10,000	10,000	10,000	10,000	10,000	10,000
Cell dimensions (Å)						
a, *b*, *c* (Å)	62.1, 62.1, 110.5	62.1, 62.1, 110.5	62.1, 62.1, 110.5	62.1, 62.1, 110.5	62.1, 62.1, 110.5	62.1, 62.1, 110.5
α, β, γ (°)	90, 90, 120	90, 90, 120	90, 90, 120	90, 90, 120	90, 90, 120	90, 90, 120
Resolution (Å)	20-1.8 (1.9-1.8)	20-1.8 (1.9-1.8)	20-1.8 (1.9-1.8)	20-1.8 (1.9-1.8)	20-1.8 (1.9-1.8)	20-1.8 (1.9-1.8)
R_{split}	0.120 (0.751)	0.119 (0.768)	0.119 (0.786)	0.118 (0.789)	0.118 (0.788)	0.120 (0.779)
CC*	0.996 (0.868)	0.996 (0.864)	0.996 (0.854)	0.996 (0.847)	0.997 (0.845)	0.996 (0.845)
$I / \sigma I$	6.0 (1.4)	6.1 (1.4)	6.0 (1.4)	6.0 (1.4)	6.0 (1.4)	6.0 (1.4)
Completeness (%)	100 (100)	100 (100)	100 (100)	100 (100)	100 (100)	100 (100)
Redundancy	139 (97)	139 (97)	138 (96)	138 (96)	138 (96)	137 (96)
Estim. twinning fraction (ML)	0.070	0.067	0.094	0.096	0.097	0.091
**Real-space refinement**						
Map resolution (Å)	20-1.8	20-1.8	20-1.8	20-1.8	20-1.8	20-1.8
f	0.15	0.15	0.15	0.15	0.15	0.15
Model/map fit (CC_{mask})	0.8364	0.8365	0.8374	0.8321	0.8387	0.8368
No. atoms						
Protein	1786	1786	1786	1786	1786	1786
Ligand/ion	21 (retinal)	21 (retinal)	21 (retinal)	21 (retinal)	21 (retinal)	21 (retinal)
Water	36	36	36	36	36	36
R.m.s. deviations						
Bond lengths (Å)	0.007	0.005	0.006	0.005	0.006	0.006
Bond angles (°)	0.986	0.905	0.944	0.873	0.954	0.954

	0.53 ps	0.56 ps	0.59 ps	0.63 ps	0.68 ps	0.74 ps
**Data collection**						
Space group	$P6_3$	$P6_3$	$P6_3$	$P6_3$	$P6_3$	$P6_3$
No. indexed images	10,000	10,000	10,000	10,000	10,000	10,000
Cell dimensions (Å)						
a, *b*, *c* (Å)	62.1, 62.1, 110.5	62.1, 62.1, 110.5	62.1, 62.1, 110.5	62.1, 62.1, 110.5	62.1, 62.1, 110.5	62.1, 62.1, 110.5
α, β, γ (°)	90, 90, 120	90, 90, 120	90, 90, 120	90, 90, 120	90, 90, 120	90, 90, 120
Resolution (Å)	20-1.8 (1.9-1.8)	20-1.8 (1.9-1.8)	20-1.8 (1.9-1.8)	20-1.8 (1.9-1.8)	20-1.8 (1.9-1.8)	20-1.8 (1.9-1.8)
R_{split}	0.120 (0.790)	0.119 (0.827)	0.120 (0.841)	0.119 (0.863)	0.121 (0.864)	0.122 (0.858)
CC*	0.996 (0.840)	0.996 (0.827)	0.996 (0.821)	0.986 (0.863)	0.986 (0.832)	0.986 (0.858)
$I / \sigma I$	6.0 (1.4)	5.9 (1.3)	5.9 (1.3)	5.9 (1.3)	5.9 (1.3)	5.9 (1.3)
Completeness (%)	100 (100)	100 (100)	100 (100)	100 (100)	100 (100)	100 (100)
Redundancy	137 (96)	136 (95)	136 (95)	136 (95)	134 (94)	133 (93)
Estim. twinning fraction (ML)	0.097	0.090	0.067	0.101	0.090	0.089
**Real-space refinement**						
Map resolution (Å)	20-1.8	20-1.8	20-1.8	20-1.8	20-1.8	20-1.8
f	0.15	0.15	0.15	0.15	0.15	0.15
Model/map fit (CC_{mask})	0.8374	0.8374	0.8347	0.8257	0.8395	0.8398
No. atoms	1786	1786	1786	1786	1786	1786
Protein	21 (retinal)	21 (retinal)	21 (retinal)	21 (retinal)	21 (retinal)	21 (retinal)
Ligand/ion	36	36	36	36	36	36
Water						
R.m.s. deviations						
Bond lengths (Å)	0.006	0.006	0.005	0.005	0.005	0.005
Bond angles (°)	0.934	0.942	0.891	0.862	0.901	0.889

Supplementary Table 5. C13-C14 torsion angle values. A list of the apparent torsion angle values for each time-delay. The dark-unrestrained structure (pdb id 6RMK) is used for the 0 fs (dark) time point.

Delay time [fs]	apparent angle [°]
0	-150.0
240	-150.5
330	-147.1
390	-141.6
430	-137.5
460	-133.6
490	-126.2
530	-131.4
560	-133.4
590	-134.2
630	-134.9
680	-134.4
740	-135.1
1000	-112.0
3000	-100.0
10000	-68

Supplementary Table 6. Frequencies of SVD right singular vectors when using only a box around retinal with a padding of 5 Å.

Vector #	Period [fs]	Frequency [cm^{-1}]	Oscillation in structures with close frequencies [cm^{-1}]	
1	11290	3		
2	607	55		
3	401	83	C18-C5-C13-C20	93+-14
			Lys216 chi4	87+-6
			Asp212 chi2	82+-6
			Asp212-Wat402	80+-9
			Tyr185 psi	81+-9
4	293	114	C18-C5-C6-C7	119+-9
			Glu204-Glu194	104+-17
			Met118 chi2	105+-12
5	232	144		
6	185	180		
7	70	478		

Supplementary Table 7. Experimental parameters of our experiment and of Nogly et al.[32]

	**Nass Kovacs et al. (this work)**	**Nogly et al[32]**
Average crystal size	25×25×2.5 µm	35×35×3.0 µm
Average path length through crystal *p*	5.9 µm<br>(transmission ~19%)	7.3 µm<br>(transmission ~13%)
Light-adaption<br>yield all-*trans* retinal	On-line<br>65-80 % (HPLC)	Off-line<br>Not determined
Offset *x* between pump laser and X-ray focus	On average 25 µm downstream<br>(20-30 µm, Supp. Fig. 13b)	50 µm downstream<br>(Supp. Fig. 13a)
Pump laser: pulse length *T*, waist diameter 2*w*	145 fs, 99 µm at $1/e^2$	100 fs, 95 µm at $1/e^2$
Pump laser energy *E*	5.9 µJ	17 µJ
Peak fluence F_0 *1<br>Peak power density I_0 *2<br><br>Power density *I* at FEL interaction zone at offset *x* *3	0.15 J cm^{-2}<br>~ 1.1 TW cm^{-2}<br><br>~ 630 GW cm^{-2}	0.48 J cm^{-2}<br>4.8 TW cm^{-2}<br>(reported 2.4 TW cm^{-2}) $$<br>~ 520 GW cm^{-2}<br>(reported 324 GW cm^{-2}) $$$
Impinging photons/retinal *4	26	12
Light loss via reflection /scattering<br>Experimental data provided	20 %<br><br>Yes	80 % ¥<br><br>No
Power density at FEL interaction zone at offset *x* #<br>incl. reduction by scattering	~510 GW cm^{-2} (20 % loss)	420 GW cm^{-2} (20 % loss)<br>100 GW cm^{-2} (80 % loss) ¥
Photons/retinal including photon loss by scattering	21 (20 % scattering loss)	10 (20 % scattering loss)<br>2 (80 % scattering loss) ¥
XFEL rep rate<br>Pump laser rep rate	10 Hz<br>10 Hz	120 Hz<br>30 HZ
Capillary inner diameter	100 µm	50 µm
Flow rate	1.9 µl/min	2.5 µl/min
Stream velocity	Measured: 4 mm/s	Not determined
M-like intermediate	collected separately at 30 Hz laser centred on X-ray focus or offset by 50 µm upstream. Reduced stream velocity (1.2 mm s^{-1}, measured)	2nd XFEL pulse after pump Laser offset *x* 50 µm downstream of X-ray focus##

*1 Peak fluence: fluence is energy *E*/area; for a Gaussian beam the maximum fluence within the beam profile occurring in the beam centre is $F_0 = E/(\pi\, w^2 / 2)$, with 2 *w* being the beam diameter (waist) at $1/e^2$ and E the total pulse energy.

*2 Peak power density: power density (intensity) is fluence /pulse length, i.e. energy per area and time. The peak power density for peak fluence F_0 and pulse length *T* is $I_0 = F_0/T$.

$$ The number given in Nogly et al.[32] (in brackets) is not correct. It was calculated assuming a flat-top ($E/\pi\,(\mathrm{FWHM})^2$) instead of Gaussian beam.

$$$ The number given in Nogly et al.[32] (in brackets) is not correct. Besides assuming a flat-top instead of a Gaussian beam it was calculated for a displacement *x* corresponding to $1/e^2$ (95/2 µm) instead of 50 µm

*3 Intensity $I(x)$ of a Gaussian beam at position *x* from the beam centre is $I(x) = I_0 * \exp(-2x^2/ w^2)$; For a pump laser offset *x* the intensity at the FEL interaction zone *I* is thus reduced by a factor of $\exp(-2x^2/w^2)$ compared to the peak intensity I_0.

*4 The number of photons per retinal N_p/N_{ret} is obtained by (1) calculating the number of photons N_p impinging a surface of $A=\pi w^2$ at an incoming intensity *I* and (2) calculating the number of retinals N_{ret} that are contained within a crystal of thickness p within the cross sectional area $A=\pi w^2$. (1) Np is determined by the laser intensity $I(x)$ at the FEL interaction zone, the pulse length *T* and the photon energy E_p by $N_p = I(x) \cdot T \cdot A / E_p$ which is the incoming energy onto the surface *A* divided by the energy of a single photon E_p =

$h \cdot c/\lambda$ where h is Planck's constant, c the speed of light and λ the wavelength. (2) N_{ret} encountered by the laser pulse illuminating a cross sectional area A is determined by the number density of retinals within the crystal $\rho_{ret} = 1.5 \cdot 10^{25}$ m^3, the average path length p through the crystal and the extend of the area A: $N_{ret} = \rho_{ret} \cdot p \cdot A$.

¥ This value is likely due to a lens effect (Supplementary Note 1).

This data set cannot be taken as a control that the ultrafast structures launch the functional photocycle, as the excitation photon densities used for the short- and long-lived intermediates differ.

Supplementary Table 8. Effect of the active-space selection on excitation energies. The excitation energies and properties of the small cluster Ret-Trp86-W402 at the S_0-min geometry were computed with different methods. For the XMCQDPT calculations, the active space is indicated as the retinal subspace and the Trp subspace. "-SAN" indicates the number of computed states. All states were included in state averaging (SA) with equal weights; all states were considered in the XMCQDPT calculations. The cc-pvdz basis set was used in all calculations.

Energy, au		tdm, au	Dipole moment, Debye				State assignment
XMCQDPT2	XMCQDPT2'		x	y	z	norm	
**(Ret12,12)-SA2**							
-1349.40951	-1349.337077		4.016	-19.859	9.028	22.18	CS
-1349.325423	-1349.260926	4.333	1.276	-1.654	2.025	2.91	(Ret)S_1
**(Ret8,8; Trp4,4)-SA2**							
-1349.39307	-1349.332083		3.909	-18.159	8.803	20.56	CS
-1349.313511	-1349.255839	4.333	1.312	-1.809	2.690	3.50	(Ret)S_1
**(Ret8,8; Trp4,4)-SA3**							
-1349.395673	-1349.339343		3.771	-17.457	8.505	19.78	CS
-1349.319575	-1349.26462	4.791	1.357	-2.673	2.893	4.17	(Ret)S_1
-1349.282703	-1349.226634	0.875	3.923	-16.624	6.520	18.29	(Ret)S_2
**(Ret8,8; Trp4,4)-SA4**							
-1349.394805	-1349.342718		3.698	-16.992	8.448	19.33	CS
-1349.320292	-1349.269197	4.933	1.243	-2.503	2.713	3.90	(Ret)S_1
-1349.280128	-1349.229164	0.837	3.614	-15.451	6.005	16.97	(Ret)S_2
-1349.249701	-1349.200794	0.132	3.472	-15.246	5.717	16.65	(Ret)S_3
**(Ret8,8; Trp4,4)-SA5**							
-1349.392327	-1349.343159		3.761	-17.185	9.411	19.95	CS
-1349.316596	-1349.268919	4.799	1.169	-1.988	3.459	4.16	(Ret)S_1
-1349.28092	-1349.236196	0.323	-0.337	-16.368	-7.030	17.82	CT(W86)
-1349.2775	-1349.230751	0.774	2.914	-14.610	4.512	15.57	(Ret)S_2
-1349.248544	-1349.203161	0.146	3.356	-14.438	6.249	16.09	(Ret)S_3
**(Ret6,6; Trp6,6) SA4**							
-1349.399608	-1349.343074		3.765	-15.614	9.538	18.68	CS
-1349.326378	-1349.275962	4.622	2.269	-7.594	4.495	9.11	(Ret)S_1
-1349.292103	-1349.245716	0.194	-2.603	-19.651	-6.572	20.89	CT(W86)
-1349.285647	-1349.234599	0.784	2.342	-6.939	4.261	8.47	(Ret)S_2
**(Ret4,4; Trp8,8) -SA3**							
-1349.409793	-1349.342863		3.447	-12.539	8.790	15.70	CS
-1349.340911	-1349.276513	5.069	2.407	-6.588	4.211	8.18	(Ret)S_1
-1349.300259	-1349.244219	0.104	-2.763	-18.839	-6.739	20.20	CT(W86)
**(Ret4,4; Trp8,8) -SA8**							
-1349.407834	-1349.350323		3.775	-14.444	9.513	17.70	CS
-1349.339775	-1349.285358	4.951	2.932	-9.654	5.623	11.55	(Ret)S_1
-1349.299894	-1349.253461	0.050	-3.036	-20.173	-5.904	21.24	CT(W86)
-1349.283311	-1349.235659	0.214	-0.788	-17.353	-7.874	19.07	CT(W)D_1
-1349.273633	-1349.220067	0.714	1.490	-3.4534	2.411	4.47	(Ret)S_2
-1349.245243	-1349.188821	0.378	3.161	-11.996	8.866	15.25	(W)S_1

-1349.228333	-1349.180733	0.021	-1.719	-18.432	-6.371	19.58	CT(W)D_2
-1349.225198	-1349.176198	0.008	1.749	-5.382	4.106	6.99	(W)S_1(Ret)S_1
**(Ret2,2; Trp2,0) -SA3**							
-1349.333884	-1349.325223		3.503	-10.752	7.888	13.79	CS
-1349.264907	-1349.256292	5.457	2.792	-6.284	3.828	7.87	(Ret)S_1
-1349.224038	-1349.223641	0.105	-4.884	-19.903	-4.875	21.07	CT(W86)
**CC2**							
-1349.374362		Osc. Str.					CS
-1349.294721		1.627	-	-	-	-	(Ret)S_1
-1349.276830		0.001	-	-	-	-	CT(W86)
-1349.256646		0.008	-	-	-	-	CT(W)D_1
-1349.241820		0.209	-	-	-	-	(Ret)S_2
-1349.215980		0.264	-	-	-	-	(Ret)S_3

Supplementary Table 9. Effect of the active-space selection on the CT excitation energies. The excitation energies and properties of the small cluster model Ret-Trp86-W402 at the CT(W86)-min geometry were computed with different methods. For the XMCQDPT calculations, the active space is indicated as the retinal subspace and the Trp subspace. "-SAN" indicates the number of computed states. All these states were included in state averaging (SA) with equal weights; all states were considered in the XMCQDPT calculations.

Energy, au			Dipole moment, Debye				State
XMCQDPT2	XMCQDPT2'	tdm, au	x	y	Z	norm	assignment
(Ret8,8; Trp4,4)-SA5							
-1349.378146	-1349.330631		4.456	-14.783	9.907	18.34	CS
-1349.309909	-1349.263099	5.205	1.704	-2.397	4.670	5.52	(Ret)S_1
-1349.297684	-1349.251476	0.131	-1.109	-16.743	-7.170	18.25	CT(W86)
-1349.282054	-1349.237318	0.575	4.092	-13.529	6.821	15.70	(Ret)S_2
-1349.251772	-1349.206719	0.346	-0.332	-14.615	-8.535	16.93	CT(W)D_1
(Ret6,6; Trp6,6)-SA4							
-1349.373457	-1349.324236		4.306	-12.737	8.668	16.00	CS
-1349.303103	-1349.260164	0.448	-3.365	-19.018	-5.568	20.10	CT(W86)
-1349.298155	-1349.250864	4.678	1.257	-0.178	3.377	3.61	(Ret)S_1
-1349.264265	-1349.217517	0.559	4.870	-15.126	7.326	17.50	S2
(Ret4,4; Trp8,8)-SA4							
-1349.373883	-1349.322548		4.142	-9.579	9.518	14.12	CS
-1349.321642	-1349.265894	0.161	-2.480	-19.704	-5.106	20.51	CT(W86)
-1349.291001	-1349.240912	4.155	1.319	-1.765	2.973	3.70	(Ret)S_1
-1349.284992	-1349.227607	1.309	-0.470	-15.843	-5.970	16.94	CT(W)D_1
(Ret2,2; Trp2,0)-SA3							
-1349.308454	-1349.307972		3.272	-6.091	7.415	10.14	CS
-1349.251221	-1349.251212	0.810	-3.443	-19.346	-5.067	20.29	CT(W86)
-1349.248455	-1349.248019	5.262	2.755	-6.435	4.091	8.11	(Ret)S_1
CC2							
-1349.362623	-	Osc. Str.	-	-	-	-	CS
-1349.296815	-	0.003	-	-	-	-	CT(W86)
-1349.285157	-	1.844	-	-	-	-	(Ret)S_1
-1349.255104	-	0.012	-	-	-	-	CT(W)D_1
-1349.240660	-	0.154	-	-	-	-	(Ret)S_2
-1349.210675	-	0.008	-	-	-	-	CT(W)D_2

Supplementary Table 10. The effect of size consistency: comparison of the XMCQDPT2 (X2) and XMCQDPT2' (X2') results for the retinal S_0-S_1 energy (eV). For the XMCQDPT calculations were performed with indicated active space. (12,12) and (2,2) consisted of only retinal MOs; (4,3) and (5,8) included MOs of the retinal and electron donors. "-SAN" indicates the number of computed states. All these states were included in state averaging (SA) with equal weights; all states were considered in the XMCQDPT calculations.

**Method**	**(12,12)-SA2**		**(2,2)-SA2**		**(4,3)-SA3***		**(8,5)-SA5****	
**Model**	**X2**	**X2'**	**X2**	**X2'**	**X2**	**X2'**	**X2**	**X2'**
RPSB	2.08	2.02	1.82	1.82	-	-	-	-
RPSB-Trp86-W402	2.29	2.07	1.84	1.84	1.89	1.89	-	-
Large cluster	3.91	3.00	2.26	2.26	-	-	2.27	2.29

* the CT(W86) state was included;

** the CT(W86), CT(Y185) and CT(W)D_1 state were included.

References

1 Bionta, M. R. *et al.* Spectral encoding of x-ray/optical relative delay. *Opt Express* **19**, 21855-21865 (2011).

2 Brehm, W. & Diederichs, K. Breaking the indexing ambiguity in serial crystallography. *Acta Crystallogr. D* **70**, 101-109 (2014).

3 White, T. A. *et al.* Recent developments in CrystFEL. *J. Appl. Crystallogr.* **49**, 680-689 (2016).

4 Vagin, A. & Teplyakov, A. MOLREP: an automated program for molecular replacement. *J. Appl. Crystallogr* **30**, 1022-1025 (1997).

5 Winn, M. D. *et al.* Overview of the CCP4 suite and current developments. *Acta Crystallogr D* **67**, 235-242 (2011).

6 Adams, P. D. *et al.* PHENIX: a comprehensive Python-based system for macromolecular structure solution. *Acta Crystallogr. D* **66**, 213-221 (2010).

7 Nango, E. *et al.* A three-dimensional movie of structural changes in bacteriorhodopsin. *Science* **354**, 1552-1557 (2016).

8 CCP4. The CCP4 suite: programs for protein crystallography. *Acta Crystallogr. D* **50**, 760–763 (1994).

9 Wickstrand, C., Dods, R., Royant, A. & Neutze, R. Bacteriorhodopsin: Would the real structural intermediates please stand up? *BBA-Gen Sub* **1850**, 536-553 (2015).

10 Bourgeois, D. New processing tools for weak and/or spatially overlapped macromolecular diffraction patterns. *Acta Crystallogr. D* **55**, 1733-1741 (1999).

11 Ursby, T. & Bourgeois, D. Improved estimation of structure-factor difference amplitudes from poorly accurate data. *Acta Crystallogr A* **53**, 564-575 (1997).

12 Emsley, P. & Cowtan, K. *Coot*: model-building tools for molecular graphics. *Acta Crystallogr D* **60**, 2126-2132 (2004).

13 Emsley, P., Lohkamp, B., Scott, W. G. & Cowtan, K. Features and development of Coot. *Acta Crystallogr D* **66**, 486-501 (2010).

14 Genick, U. K. *et al.* Structure of a protein photocycle intermediate by millisecond time-resolved crystallography. *Science* **275**, 1471-1475 (1997).

15 Genick, U. K. Structure-factor extrapolation using the scalar approximation: theory, applications and limitations. *Acta Crystallogr. D* **63**, 1029-1041 (2007).
16 Pande, K. *et al.* Femtosecond structural dynamics drives the trans/cis isomerization in photoactive yellow protein. *Science* **352**, 725-729 (2016).
17 Schmidt, M., Rajagopal, S., Ren, Z. & Moffat, K. Application of singular value decomposition to the analysis of time-resolved macromolecular X-ray data. *Biophys J* **84**, 2112-2129 (2003).
18 Afonine, P. V., Headd, J. J., Terwilliger, T. & Adams, P. D. New tool: phenix.real_space_refine. *Computational Crystallography Newsletter* **4**, 43-44 (2013).
19 Lomb, N. R. Least-squares frequency-analysis of unequally spaced data. *Astrophys. Space Sci* **39**, 447-462 (1976).
20 Scargle, J. D. Studies in astronomical time series analysis. II - Statistical aspects of spectral analysis of unevenly spaced data. *ApJ* **263**, 835-853 (1982).
21 Barends, T. R. M. *et al.* Direct observation of ultrafast collective motions in CO myoglobin upon ligand dissociation. *Science* **350**, 445-450 (2015).
22 Scherrer, P., Mathew, M. K., Sperling, W. & Stoeckenius, W. Retinal Isomer Ratio in Dark-Adapted Purple Membrane and Bacteriorhodopsin Monomers. *Biochemistry-Us* **28**, 829-834 (1989).
23 Muders, V. *et al.* Resonance Raman and FTIR spectroscopic characterization of the closed and open states of channelrhodopsin-1. *FEBS Letters* **588**, 2301-2306 (2014).
24 *Springer Handbook of Lasers and Optics, Editor F. Träger*(Springer, 2007).
25 Debus, B. *et al.* A multivariate curve resolution approach to separate UV–vis scattering and absorption contributions for organic nanoparticles. *Chemom. Intell. Lab. Sys.* **160**, 72-76 (2017).
26 Christiansen, O., Koch, H. & Jorgensen, P. The second-order approximate coupled cluster singles and doubles model CC2. *Chem Phys Chem* **243**, 409-418 (1995).
27 Ahlrichs, R., Bar, M., Haser, M., Horn, H. & Kolmel, C. Electronic structure calculations on workstation computers: The program system turbomole. *Chem Phys Chem* **162**, 165-169 (1989).
28 Gozem, S., Luk, H. L., Schapiro, I. & Olivucci, M. Theory and Simulation of the Ultrafast Double-Bond Isomerization of Biological Chromophores. *Chem Rev* **117**, 13502-13565 (2017).
29 Granovsky, A. A. Extended multi-configuration quasi-degenerate perturbation theory: The new approach to multi-state multi-reference perturbation theory. *J Chem Phys* **134** (2011).
30 Schmidt, B. *et al.* Excited-state dynamics of bacteriorhodopsin probed by broadband femtosecond fluorescence spectroscopy. *BBA-Bioenergetics* **1706**, 165-173 (2005).
31 Prokhorenko, V. I., Halpin, A., Johnson, P. J. M., Miller, R. J. D. & Brown, L. S. Coherent control of the isomerization of retinal in bacteriorhodopsin in the high intensity regime. *J Chem Phys* **134** (2011).
32 Nogly, P. *et al.* Retinal isomerization in bacteriorhodopsin captured by a femtosecond x-ray laser. *Science* **361**, eaat0094 (2018).
